# Mid-term changes in cognitive functions in patients with atrial fibrillation: a longitudinal analysis of the Swiss-AF cohort

**DOI:** 10.3389/fcvm.2023.1212587

**Published:** 2023-08-02

**Authors:** Alexandra S. Wueest, Priska Zuber, Michael Coslovsky, Nikki Rommers, Nicolas Rodondi, Baris Gencer, Giorgio Moschovitis, Maria Luisa De Perna, Juerg H. Beer, Tobias Reichlin, Philipp Krisai, Anne Springer, David Conen, Annina Stauber, Andreas S. Mueller, Rebecca E. Paladini, Michael Kuhne, Stefan Osswald, Andreas U. Monsch, Leo H. Bonati

**Affiliations:** ^1^ University Department of Geriatric Medicine FELIX PLATTER, Memory Clinic, Basel, Switzerland; ^2^ Clinic for Anaesthesia, Intermediate Care, Prehospital Emergency Medicine and Pain Therapy, University Hospital Basel, Basel, Switzerland; ^3^ Division of Cognitive Neuroscience, Faculty of Psychology, University of Basel, Basel, Switzerland; ^4^ Cardiology Division, Department of Medicine, University Hospital Basel, Basel, Switzerland; ^5^ Cardiovascular Research Institute Basel (CRIB), Basel, Switzerland; ^6^ Department of Clinical Research, University of Basel, University Hospital Basel, Basel, Switzerland; ^7^ Institute of Primary Health Care (BIHAM), University of Bern, Bern, Switzerland; ^8^ Department of General Internal Medicine, Inselspital, Bern University Hospital, University of Bern, Bern, Switzerland; ^9^ Division of Cardiology, Ente Ospedaliero Cantonale (EOC), Cardiocentro Ticino Institute, Regional Hospital of Lugano, Lugano, Switzerland; ^10^ Department of Medicine, Cantonal Hospital of Baden and Molecular Cardiology, University Hospital of Zürich, Zürich, Switzerland; ^11^ Department of Cardiology, Inselspital, Bern University Hospital, University of Bern, Bern, Switzerland; ^12^ Population Health Research Institute, McMaster University, Hamilton, ON, Canada; ^13^ Department of Cardiology, Triemli Hospital Zürich, Zürich, Switzerland; ^14^ Department of Neurology, University of Basel, University Hospital Basel, Basel, Switzerland; ^15^ Department of Research, Reha Rheinfelden, Rheinfelden, Switzerland

**Keywords:** atrial fibrillation, cognitive function, longitudinal cohort study, Swiss-AF, practice effect

## Abstract

**Background:**

Longitudinal association studies of atrial fibrillation (AF) and cognitive functions have shown an unclear role of AF-type and often differ in methodological aspects. We therefore aim to investigate longitudinal changes in cognitive functions in association with AF-type (non-paroxysmal vs. paroxysmal) and comorbidities in the Swiss-AF cohort.

**Methods:**

Seven cognitive measures were administered up to five times between 2014 and 2022. Age-education standardized scores were calculated and association between longitudinal change in scores and baseline AF-type investigated using linear mixed-effects models. Associations between AF-type and time to cognitive drop, an observed score of at least one standard deviation below individual's age-education standardized cognitive scores at baseline, were studied using Cox proportional hazard models of each cognitive test, censoring patients at their last measurement. Models were adjusted for baseline covariates.

**Results:**

2,415 AF patients (mean age 73.2 years; 1,080 paroxysmal, 1,335 non-paroxysmal AF) participated in this Swiss multicenter prospective cohort study. Mean cognitive scores increased longitudinally (median follow-up 3.97 years). Non-paroxysmal AF patients showed smaller longitudinal increases in Digit Symbol Substitution Test (DSST), Cognitive Construct Score (CoCo)and Trail Making Test part B (TMT-B) scores vs. paroxysmal AF patients. Diabetes, history of stroke/TIA and depression were associated with worse performance on all cognitive tests. No differences in time to cognitive drop were observed between AF-types in any cognitive test.

**Conclusion:**

This study indicated preserved cognitive functioning in AF patients, best explained by practice effects. Smaller practice effects were found in non-paroxysmal AF patients in the DSST, TMT-B and the CoCo and could indicate a marker of subtle cognitive decline. As diabetes, history of stroke/TIA and depression—but not AF-type—were associated with cognitive drop, more attention should be given to risk factors and underlying mechanisms of AF.

## 1. Introduction

Atrial fibrillation (AF) is the most common cardiac arrhythmia affecting people of all ages (1, 2). As its incidence and prevalence increase with age, it is estimated that about 17.9 million adults aged over 55 years in 2060 will experience AF in the European Union (3). Adjusting for age and predisposing conditions, AF has been associated with an increased risk of stroke (4), heart failure (5), myocardial infarction (MI) (6) and death (7, 8). In addition, numerous previous studies, including several meta-analyses, provide growing evidence of an association between AF and cognitive decline in the absence of clinically overt previous stroke (9, 10).

Independent from other risk factors, a correlative study described an association between AF and a greater cognitive decline over 20 years in people with AF compared to people without AF (11). Concerning the affected cognitive domain, AF has been associated with poorer performance and longitudinal decline in executive functions (12). However, the current knowledge status is insufficient since the studies suffer from large heterogeneity in methods.

First, studies differ in methodological aspects such as sample size, duration of follow-up periods and statistical methods, as well as with respect to the assessments of cognitive performance (1, 13). Second, not all studies systematically adjusted for known confounders in multivariable models since AF and cognitive decline share multiple risk factors and comorbidities (14). Finally, it has been shown that next to increasing age, the prevalence of heart failure, hypertension, diabetes, coronary artery, and cerebrovascular diseases increases with progression from paroxysmal to non-paroxysmal (persistent or permanent) AF subtype (15). Although the progression of AF and the development of cognitive decline share common risk factors (1), the association of AF and its subtypes with change in cognitive function as well as the possible effects of comorbidities have not been investigated. Additionally, it is unknown if non-paroxysmal AF, characterized as persistent or permanent and with increased symptom severity, is more involved in change of cognitive function than paroxysmal AF (16).

Thus, longitudinal studies are needed that investigate the association between AF subtype and longitudinal change in cognitive function. In the Swiss-AF Cohort, we aim to investigate the association of AF subtype and change in cognitive functions over time in patients with AF, while accounting for comorbidities, using an extensive assessment of cognitive functioning with validated tests assessing multiple cognitive domains. Specifically, we aim to (1) describe the development of longitudinal cognitive functioning in a typical Swiss population of patients with AF; (2) assess whether changes of cognitive functions are associated with AF-type (i.e., paroxysmal or non-paroxysmal) and (3) describe the frequency of cases of cognitive drop over time, defined as an observed score of at least one standard deviation (SD) below the individual's age-education standardized cognitive test scores at baseline and assess the association with AF-type and comorbidities.

## 2. Materials and methods

### 2.1. Study sample

The Swiss Atrial Fibrillation Study (Swiss-AF; NCT02105844) is an ongoing prospective, multicenter, observational, national cohort study in primarily elderly individuals with AF focusing on the interrelationships of AF and AF progression with structural and functional brain damage over time. Details about the sampling method and selection process are described elsewhere (17–19). Briefly, a total of 2,415 subjects with a history of AF at baseline (BL) (17) were recruited by comprehensive screening of in- and outpatients in 14 participating centers in Switzerland between 2014 and 2017 and by contacting general practitioners in the area. Main inclusion criteria were age 65 years or older (with the exception of additional 315 patients aged between 37 and 65 years, which were enrolled to assess socio-economic aspects of AF in the working population) and presence of either paroxysmal or non-paroxysmal AF according to the guidelines of the European Society of Cardiology (20). 87% of patients included in the present analysis were 65 years or older (*n* = 2,100). Paroxysmal AF was defined as self-terminating AF lasting <7 days, did not require cardioversion and was documented at least twice within the past 5 years (20). Persistent AF was defined as AF sustained for at least 7 days and/or AF requiring cardioversion, documented within the past 5 years by electrocardiography (ECG) or rhythm monitoring devices (20). Permanent AF was defined as AF in which cardioversion therapy failed or was not attempted (20). For the current study, participants were categorized as having paroxysmal and non-paroxysmal (including persistent and permanent) AF. Details about the assessment of AF-type are described in a previous publication (21). Briefly, the local study investigator determined AF-type during the baseline visit based on all available clinical patient data over the years before enrollment, documented by medical records, ECG, and/or rhythm monitoring device. We excluded patients who were unable to provide informed consent, had any acute illness within the last 4 weeks or indicated only secondary, reversible episodes of AF (e.g., after cardiac surgery or severe sepsis). Regarding the integrity of cognitive abilities, no further requirements were defined since we aimed to establish a representative large sample of elderly patients with diagnosed AF.

### 2.2. Study procedures

Trained study personnel collected all data in a standardized manner. Specifically, a training video for the cognitive assessment was made available for all investigators at all sites. At enrollment, participants underwent a clinical examination and cognitive assessment. Detailed information on personal characteristics, risk factors and comorbidities were obtained through standardized case report forms (CRF). The study was approved by the local Ethics Committee–Ethikkommission Nordwest- und Zentralschweiz (EKNZ number: PB_2016_00793) and conducted in accordance with the Declaration of Helsinki. All subjects gave written informed consent before participation. In the current analysis, we considered patients who were administered at least one cognitive test at baseline only or at any of the planned follow-up visits within 4 years (+50 days). A total of 109 follow-up 4 visits were excluded having taken place >50 days later than planned. The data for this analysis reflects the status of the Swiss-AF data base as on May 13, 2022, in which all patients were enrolled in the cohort for at least 4 years.

### 2.3. Assessment of cognitive function

The cognitive test battery consisted of five validated, widely used cognitive tests administered at each follow-up. The test battery included the Montreal Cognitive Assessment (MoCA) (22), Trail Making Test Part A (TMT-A) (23, 24) Trail Making Test Part B (TMT-B) (TMT results calculated as number of correct connections per second) (23, 24), Semantic Fluency test (SF) (25) and the Digit Symbol Substitution Test (DSST) (26, 27), described in detail elsewhere (19). All cognitive tests were administered in a paper-pencil format or, during the coronavirus disease 2019 (COVID-19) pandemic, in part (i.e., MoCA and SF) by telephone. Cognitive tests were administered in the main national languages of Switzerland, depending on patient's mother tongue (i.e., 72.4% German, 11.9% French and 10.2% Italian), except for the TMT and the DSST, which are language-independent tests. Supplementary Table S1 in the supplement provides an overview of the neuropsychological test battery, which consists of 17 outcome variables. The standard MoCA total score was calculated (28). MoCA results administered by telephone (*n* = 221) were discarded, since these results do not reflect the in-person test. Additionally, two derived cognitive measures were used, i.e., the ratio TMT-B / TMT-A and the Cognitive Construct (CoCo) derived from the total of 17 items comprised in the five validated neuropsychological tests used in the Swiss-AF cohort study. A previous study of the group has shown that using the CoCo score increased measurement sensitivity and allows to detect subtle changes in cognitive function (19). Cognitive drop was defined as an observed score of at least one SD below the individual's age-education standardized cognitive test scores at baseline (29). All scores of the cognitive tests were standardized by age and years of education, which were used in all analysis to describe within-patient trajectories over time from the first visit and to compare cognitive functioning stratified by AF-type. For all cognitive tests, positive values represent better results.

### 2.4. Additional variables

Socio-demographic measures including age, sex and education (years) were obtained through standardized CRFs. Health behavior including smoking (yes/no) was collected from patients' self-reports. Chronic disease included history of diabetes (yes/no), history of hypertension (yes/no), history of stroke or transient ischemic attack (TIA) (yes/no) were self-reported from the patients as well as verified by medical records. Use of medication for cardiovascular disease (anticoagulants drugs) (yes/no) and glomerular filtration rate (GFR) (milliliter/minute) was collected from medical reports. Depression (yes/no) was assessed with the Geriatric Depression Scale (GDS; range 0–15, a value ≥5 indicating depression) (30).

### 2.5. Statistical analyses

Available information for each patient from baseline until follow-up 4, or until loss to any follow-up was used. Missing data were not imputed and patients who did not have any cognitive assessment over the years (none) and with missing baseline covariates (*n* = 57) were excluded from the analysis. For 53 patients GFR was missing, other 4 patients had missing values for the other baseline covariates. In the longitudinal analysis, we included all measures of a patient, so it was also possible to include a patient if the baseline measurement was missing, but cognitive assessments at different time points were available. Due to the exploratory rather than confirmatory nature of the study, all results are presented as estimated effect sizes with 95% confidence intervals (CI). Continuous baseline characteristics are described via the mean and standard deviation or, if strongly skewed, using the median and interquartile range (IQR); frequencies and percentages are listed for categorical characteristics. All analyses were performed using the statistical software R version 4.2.2. The association between AF-type and evolution of cognitive functioning was assessed using linear mixed effects models with the age-education standardized cognitive score (see description in Text S1) as outcome and the time since first measurement, AF subtype and the interaction between them as fixed effects. To acknowledge the possibility of a practice effect, which seemed largest between the first and second measurement (28), we added an additional variable, indicating whether it was the first measurement (yes/no). Time since first measurement was added to the model as a random slope, and random intercepts for each patient, nested within study center, were included. Separate models were constructed for each of the above-mentioned outcome measures. Model diagnostics were performed by examination of residuals. The association between AF-type and relevant cognitive drop was assessed using Cox proportional hazard models stratified by study center. Patients were censored at the last measured value (i.e., the last visit during which the cognitive test was performed) in case of death or drop out, or their administrative fourth follow-up, in case the patient had more follow-up visits available. Only the first drop in cognition per patient was considered for analysis. The analyses assumed that missing cognitive assessments were not associated with the event of cognitive drop (see section sensitivity analyses). The proportional hazard assumption was tested and the Schoenfeld residuals were visually inspected as model diagnostics. We found no strong deviations from this assumption for any of the models.

### 2.6. Subgroup, sensitivity and posthoc analyses

To assess subgroup effects by sex, history of stroke, smoking status, or for patients with and without diabetes, hypertension, or depression, an interaction between each covariate of interest and AF subtype for each model was tested. For covariates with a signal of an interactive effect with AF subtype (interaction term *p*-value <0.05), we repeated the analyses in each level of the covariate (e.g., smokers vs. non-smokers). The robustness of our previous assumptions was tested in several sensitivity analyses. First, we evaluated the assumption of the linear development of cognitive functioning over time, using scatter plots with local estimated scatterplot smoothing (LOESS) curves fit to describe the development over time for each of the cognitive tests. Second, we evaluated the appropriateness of the definition of cognitive drop at >1 SD decrease by using an arbitrary >1.5 SD for relevant cognitive drop and comparing the results. Lastly, the potential extent of an attrition bias was investigated by repeating the analyses using the analysis set which included all patients who were still part of the cohort after follow-up 4, and who were able to perform at least one of the cognitive tests at follow-up 4 (SF) with the patients who dropped out before or did not do cognitive tests at follow-up 4. Furthermore, taking losses to follow-up as (potentially) informative censoring events, we repeated the analysis using linear mixed effects models incorporating patients' inverse probability of censoring weights (IPCW) and comparing the results with the models from the main analysis. Due to the consistent relatively high estimates of three covariates (i.e., history of diabetes, depression, and history of stroke/TIA) on all cognitive tests we performed the above-described linear mixed effects as *post hoc* analyses to assess whether these variables were associated with an increase over time on one representative cognitive score (i.e., MoCA). The association of these covariates with cognitive drop are already explained in the above-described Cox-models.

## 3. Results

### 3.1. Patients' characteristics

**Figure 1:**
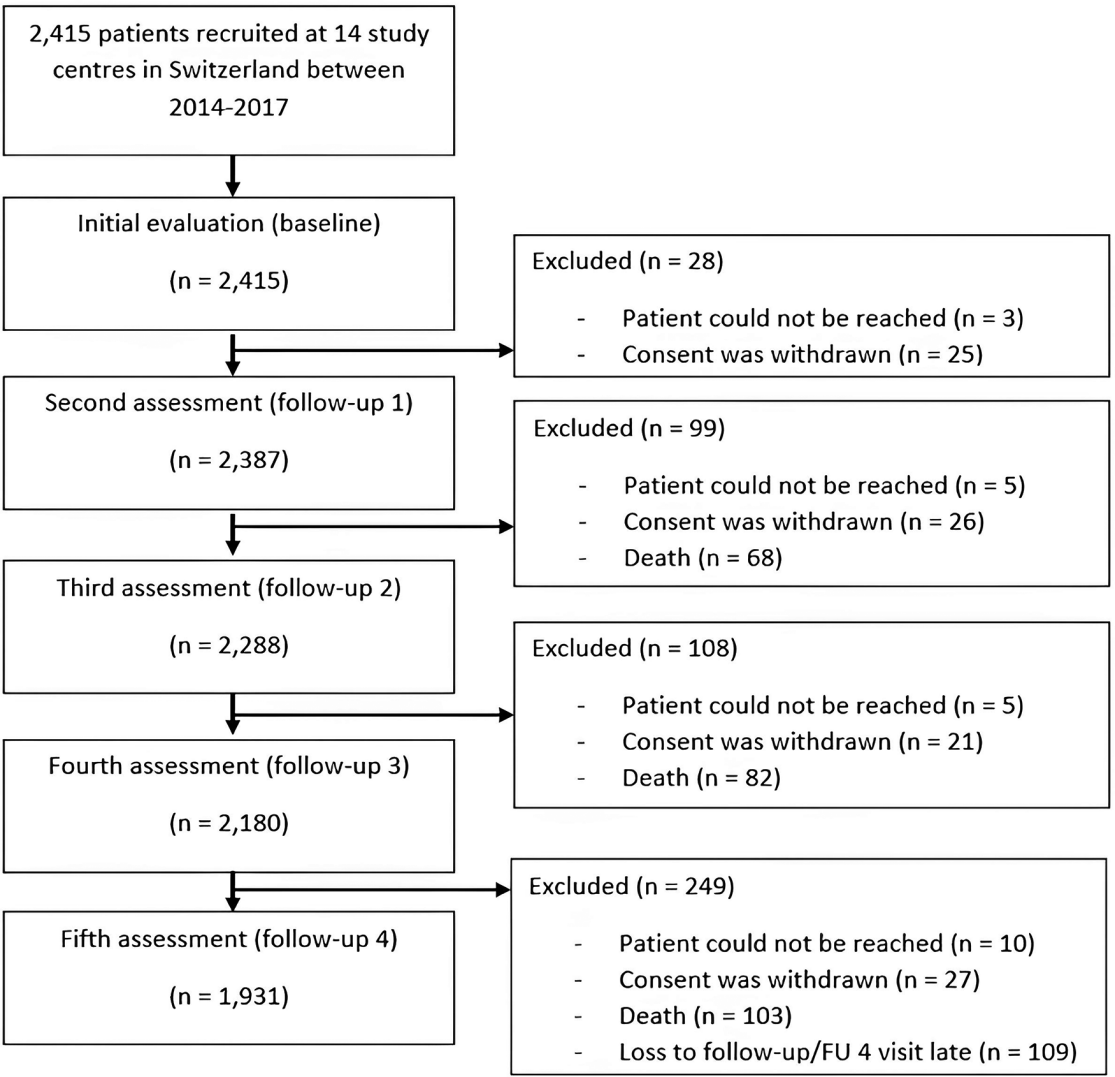
Study flow chart.

Baseline characteristics are shown in Table 1. At study enrollment, mean age was 73.2 ± 8.4 years, 27.4% of participants were women, and 90.4% were anticoagulated. 44.7% of the 2,415 included participants had paroxysmal AF, whereas 55.3% had non-paroxysmal AF. For 2,358 participants' data on all cognitive measures was available at baseline. The median follow-up was 3.97 years. Supplementary Table S2 presents the number of missing tests per visit. The number of missing test results gradually increased over the follow-up visits, being greater than 46% at follow-up for all cognitive tests except for SF. An overview of the number of cognitive tests completed per patient during each visit is available in Supplementary Table S3. The number of performed cognitive assessment for MoCA, TMT-A, TMT-B, SF and DSST as well as for the two derived scores CoCo and TMT-B/TMT-A at baseline and at follow-up 1–4 are provided in Table 2. The main reason for dropouts between BL—follow-up 3 were (1) patient could not be reached (*n* = 23); (2) consent was withdrawn (*n* = 99); (3) death (*n* = 253); (4) loss to follow-up/follow-up 4 visit >50 days after 4-year mark (*n* = 109). For a detailed overview, see Figure 1 and Supplementary Table S4. The reasons why cognitive assessments were missed or excluded from the analysis were a constrained test situation or motivation, present incident of participant (e.g., health issue, emotional or mental incident), cognitive inability to perform the test, due to dropout or death or errors of administration by the examiner.

**Table 1:** Baseline characteristics.

	Overall	Paroxysmal	Non-paroxysmal
*n*	2,415	1,080	1,335
Age (years)	73.2 (8.4)	72.5 (8.5)	73.9 (8.3)
Sex = female (%)	662 (27.4)	345 (31.9)	317 (23.7)
Education groups (%)			
Basic*	288 (11.9)	130 (12.0)	158 (11.9)
Middle*	1,197 (49.6)	531 (49.2)	666 (50.0)
Advanced*	926 (38.4)	418 (38.7)	508 (38.1)
Education (years)	12.93 (3.2)	13.05 (3.3)	12.84 (3.2)
History of stroke/TIA (%)	480 (19.9)	235 (21.8)	245 (18.4)
History of diabetes (%)	422 (17.5)	173 (16.0)	249 (18.7)
History of hypertension (%)	1,691 (70.0)	721 (66.8)	970 (72.7)
Depression (%)	200 (8.3)	84 (7.8)	116 (8.7)
Oral anticoagulation medication (%)	2,182 (90.4)	932 (86.3)	1,250 (93.6)
GFR (ml/min.)	59.29 (19.1)	60.98 (19.9)	57.94 (18.3)
Active smoker (%)	175 (7.3)	87 (8.1)	88 (6.6)

Data are presented as mean (± SD) or counts (percentages).

GFR, glomerular filtration rate; min., minutes; ml, milliliter; TIA, transient ischemic attack.

* Basic education: ≤6 years (less than compulsory education curriculum); middle education: 6 to ≤12 years (high school or similar); advanced education: ≥12 years (college or university degree).

**Table 2:** Cognitive scores over the course of the study.

	Baseline*		Follow-up 1		Follow-up 2		Follow-up 3		Follow-up 4	
	*n*	Mean (SD)	*n*	Mean (SD)	*n*	Mean (SD)	*n*	Mean (SD)	*n*	Mean (SD)
MoCA	2,402	24.9 (3.2)	2,108	25.5 (3.2)	1,889	25.9 (3.3)	1,531	26.3 (3.2)	990	26.5 (3.2)
SF	2,408	18.9 (5.4)	2,116	19.5 (5.7)	1,902	19.7 (5.8)	1,688	20.1 (6.1)	1,460	20.2 (6.4)
TMT-A	2,393	0.5 (0.2)	2,106	0.6 (0.2)	1,891	0.6 (0.2)	1,533	0.6 (0.2)	991	0.6 (0.2)
TMT-B	2,380	0.2 (0.1)	2,097	0.2 (0.1)	1,879	0.2 (0.1)	1,524	0.2 (0.1)	982	0.3 (0.1)
TMT B/A	2,380	0.4 (0.2)	2,097	0.4 (0.2)	1,876	0.4 (0.2)	1,523	0.4 (0.2)	982	0.4 (0.1)
DSST	2,396	43.6 (14.3)	2,096	45.1 (14.9)	1,871	45.9 (15)	1,520	47.6 (15.2)	987	48.2 (15.6)
CoCo	2,359	0 (0.5)	2,080	0 (0.5)	1,850	0.1 (0.6)	1,502	0.1 (0.6)	976	0.2 (0.6)

Data are presented as mean (± SD) or counts (percentages).

CoCo, Cognitive construct; DSST, Digit Symbol Substitution Test; MoCA, Montreal Cognitive Assessment; SF, Semantic Fluency Test, animals; TMT-A, Trail Making Test A; TMT-B, Trail Making Test B (number of correct connections per second); TMT B/A, ratio of Trail Making Test B/Trail Making Test A.

* *n *= 2,415.

### 3.2. Development of cognitive functioning over time

**Figure 2:**
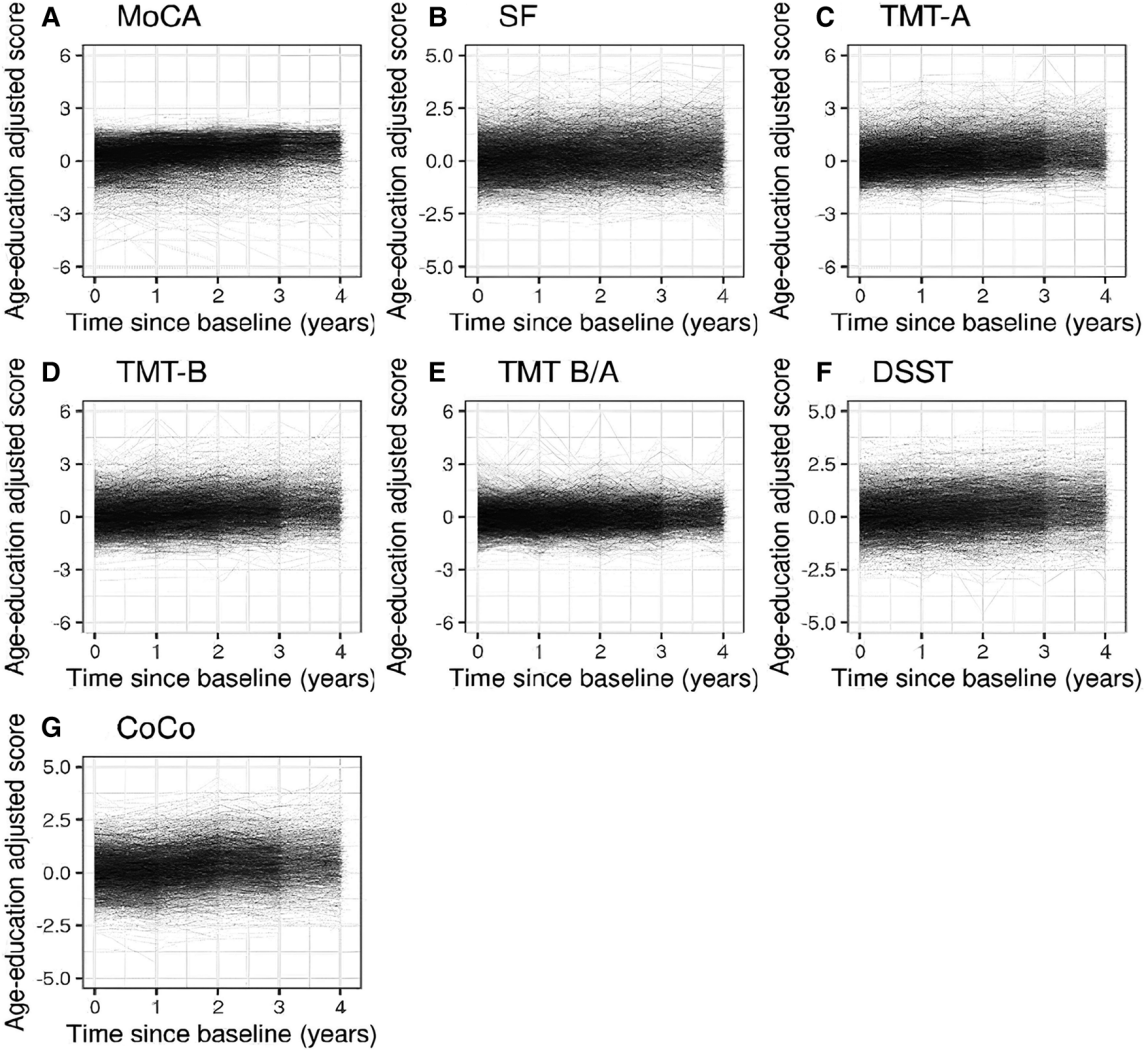
Spaghetti plots showing the evolution of the cognitive functioning over time (until follow-up 4) for each of the age-education standardized cognitive measures for all AF patients.

The mean score for each of the cognitive tests increased over time, which is displayed in Table 2 and visualized in Figure 2. Development over time for AF-type is displayed in Supplementary Figures S1, S2.

### 3.3. Cognitive development as a function of time and AF type

**Figure 3:**
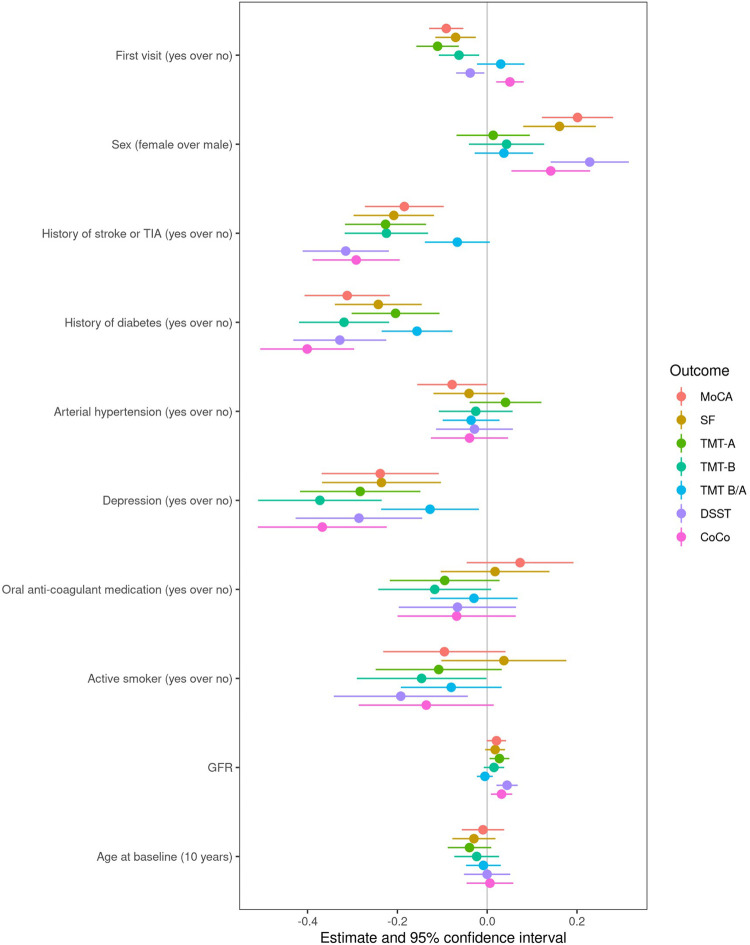
Forest plot visualizing the estimates and 95% CIs for the covariates in the linear mixed effects models of change in cognitive scores over time.

Based on visual inspection of the results, including fitting a LOESS curve for each endpoint (Supplementary Figure S3), we accepted the assumption of a linear development of cognitive functioning over time. The age-education standardized score increased over time for the MoCA, SF, TMT-A, TMT-B, DSST and CoCo. Undertaking a cognitive test for the first time was estimated to differ from the mean of repeated undertakings by −0.09 units 95% CI [−0.13, −0.05] for the MoCA score, −0.07 units [−0.12, −0.03] for the SF score, −0.11 units [−0.16, −0.06] for the TMT-A score; −0.06 units [−0.11, −0.02] for the TMT-B score; −0.04 units [−0.07, −0.01] for the DSST score and 0.05 units [0.02, 0.08] for the CoCo score. The 95% CI for the interaction between AF-type and time laying completely below 0, suggested that the development in standardized TMT-B, DSST and CoCo score depended on AF-type, after adjustment for all covariates in the model. The point estimate of the interaction term suggested a smaller yearly increase in the DSST (0.07 compared to 0.09), CoCo score (0.11 compared to 0.14) and TMT-B (0.10 compared to 0.13) in patients with non-paroxysmal AF compared to patients with paroxysmal AF. For the other remaining cognitive tests, we found no interaction effect between AF-type and time, suggesting that the development in age-education standardized of the MoCA, SF, TMT-A and the TMT-B/TMT-A scores over time is not associated with AF-type. An overview is presented in Table 3. The covariates depression, history of stroke or TIA and diabetes appeared to be associated with worse scores for all 7 standardized cognitive outcomes (Figure 3). An overview of the estimates and corresponding 95% CI for all variables in the linear fixed effects model describing the association between AF-type and the development of cognitive functioning in each cognitive test is presented in Supplementary Tables S5–S11.

**Table 3:** Estimates with 95% CI for all 7 cognitive tests. The results for each covariate represent the effect after adjusting for all other variables in the model.

Test	Variable	Estimate	95% CI
MoCA	AF-type (non-paroxysmal over paroxysmal)	−0.06	[−0.14, 0.01]
	Time (years)	0.09	[0.06, 0.12]
	AF-type*Time Interaction	−0.01	[−0.04, 0.01]
SF	AF-type (non-paroxysmal over paroxysmal)	−0.03	[−0.10, 0.05]
	Time (years)	0.03	[0.00, 0.05]
	AF-type*Time Interaction	0.01	[−0.02, 0.03]
TMT-A	AF-type (non-paroxysmal over paroxysmal)	−0.06	[−0.14, 0.02]
	Time (years)	0.09	[0.07, 0.12]
	AF-type*Time Interaction	−0.01	[−0.04, 0.01]
TMT-B	AF-type (non-paroxysmal over paroxysmal)	−0.03	[−0.11, 0.05]
	Time (years)	0.10	[0.07, 0.13]
	AF-type*Time Interaction	−0.03	[−0.05, −0.00]
TMT-B/A	AF-type (non-paroxysmal over paroxysmal)	0	[−0.07, 0.07]
	Time (years)	0.02	[−0.01, 0.05]
	AF-type*Time Interaction	−0.01	[−0.04, 0.01]
DSST	AF-type (non-paroxysmal over paroxysmal)	−0.07	[−0.15, 0.01]
	Time (years)	0.07	[0.06, 0.08]
	AF-type*Time Interaction	−0.02	[−0.04, −0.00]
CoCo	AF-type (non-paroxysmal over paroxysmal)	−0.05	[−0.13, 0.03]
	Time (years)	0.11	[0.09, 0.13]
	AF-type*Time Interaction	−0.03	[−0.04, −0.01]

CoCo, Cognitive construct; DSST, Digit Symbol Substitution Test; MoCA, Montreal Cognitive Assessment; SF, Semantic Fluency Test, animals; TMT-A, Trail Making Test A; TMT-B, Trail Making Test B (number of correct connections per second); TMT B/A, ratio of Trail Making Test B/Trail Making Test A.

### 3.4. Cognitive drop and the association with AF type

**Figure 4:**
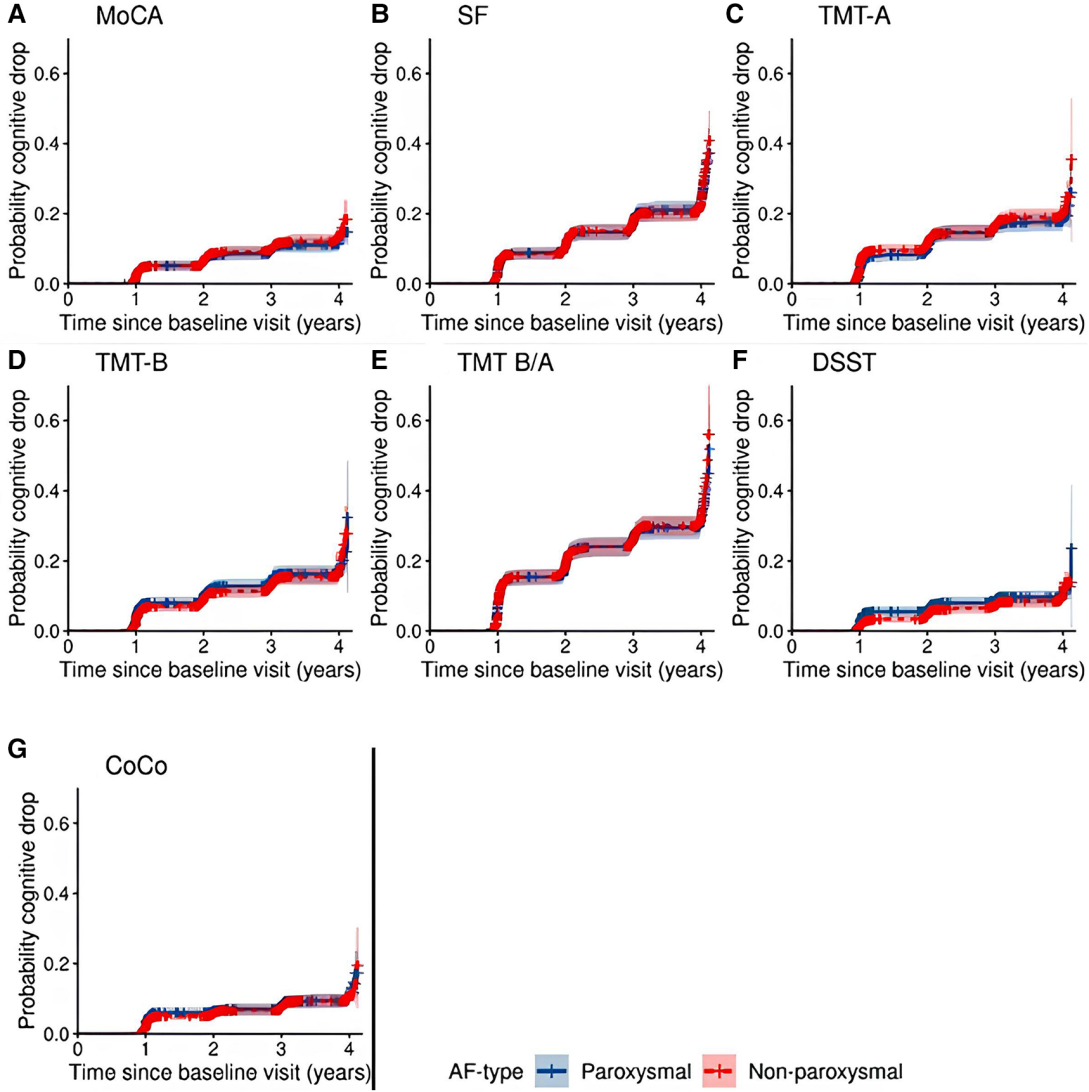
Cumulative probability of cognitive drop with 95% confidence interval according to all 7 cognitive tests by AF-type.

The threshold of cognitive drop >1 SD of the standardized baseline distribution was crossed by a total of 228 patients for MoCA, 472 for SF, 352 for TMT-A, 310 for TMT-B, 591 for TMT-B/TMT-A, 184 for DSST, and 190 for CoCo across all follow-up visits. Supplementary Table S12 presents an overview of the number of patients who crossed the threshold of 1 SD lower than the first test result per visit. AF-type did not appear to be associated with the hazard (HR) for cognitive drop for any of the cognitive tests in the model adjusted for all covariates. Figure 4 shows the probability of cognitive drop by AF-type, unadjusted for other variables, and Supplementary Tables S13–S19 report the HRs and corresponding confidence intervals of all variables in the model for each cognitive test.

### 3.5. Subgroup and sensitivity analyses

There was no association between AF-type and cognitive development and decline in the different subgroups with and without a history of diabetes, depression, or stroke/TIA, as well as by sex and smoking status. Only for a history of hypertension, we found a signal for a subgroup effect in the linear mixed models for MoCA, TMT-B, DSST, and CoCo (Supplementary Table S21 in the supplement). In the subgroup of patients without hypertension, we found a smaller increase in age-education standardized cognitive scores in patients with non-paroxysmal AF compared to patients with paroxysmal AF. The results of these models for the MoCA, TMT-B, DSST and CoCo are shown in Supplementary Tables S22–S25. In the Cox models, we did not find signals for a potential subgroup effect.

A sensitivity analysis to explore how attrition bias could confound the results was conducted using only patients who completed all five visits: baseline and four follow-ups. A comparison of these patients and the patients who dropped-out of the study before follow-up 4 is shown in Supplementary Table S20. The different sensitivity analyses (Supplementary Figure S3, Tables S26–S31), despite not completely alleviating the risk of attrition bias, suggested that in the observed time frame the effect of attrition on estimates is rather small, since the results of the analyses were similar when using patients who had all visits compared to the whole sample. Post hoc analyses indicated an interaction between the time and history of stroke or TIA at baseline, suggesting a smaller annual increase in MoCA score in patients with history of stroke or TIA compared to patients without history of stroke or TIA and are displayed in Supplementary Tables S32–S34. The association between each covariate and cognitive drop can be found in Supplementary Table S13. As post-hoc analyses we compared cognitive drop in the MoCA test scores according to history of stroke or TIA (Supplementary Figure S4), depression (Supplementary Figure S5), and diabetes (Supplementary Figure S6) in the supplement. These analyses showed a higher cumulative incidence of cognitive drop over time among patients with history of stroke or TIA [HR: 1.43, CI (1.06, 1.93)] and among patients with depression [HR: 1.26, CI (0.79, 2.00)].

## 4. Discussion

This large community-based cohort study of elderly patients with AF in Switzerland aimed at describing the development of cognitive functioning over time, its association with AF-type (paroxysmal or non-paroxysmal) and accumulation of cases of cognitive drop over time and across AF-type and comorbidities. The results showed a longitudinal increase in all mean cognitive scores with a smaller increase in DSST, TMT-B and CoCo score over time in patients with non-paroxysmal AF compared to patients with paroxysmal AF. No differences in the rate of accumulation of cognitive drop were observed between AF-types in any cognitive measure. The presence of diabetes, history of stroke/TIA and depression was associated with worse cognitive performance on all cognitive measures.

Despite growing evidence of an association between AF and cognitive decline in the absence of clinically overt previous stroke, suggested by previous work (9, 10), we found an increase in standardized mean scores of each cognitive test over time in a Swiss cohort of AF patients. Whereas the large number of AF patients and the avoidance of ceiling effects by performing validated cognitive testing on multiple domains in our study support the robustness of our results, at least three effects might explain our findings. First, at 90%, our sample had a relatively high proportion of patients that used oral anticoagulants. As oral anticoagulants act as prevention for stroke (31, 32), which has been associated with cognitive decline (33, 34), the protective effects of anticoagulants might ameliorate the risk for developing cognitive decline in our sample. Whether a broader use of oral anticoagulation, or more intense anticoagulation in some patients are beneficial in preventing cognitive decline in AF patients' needs to be addressed in future studies. Second, longitudinal studies may be prone to attrition bias, indicating that participants who are most likely to remain in the study tend to be the healthiest, best educated, wealthiest, and have the highest scores on cognitive tests, whereas ill participants are less likely to return for study visits (35). Under the limitation of our available data, sensitivity analysis using IPCW and comparison of full and dropout data sets suggested no strong evidence of attrition bias, at least in the observed period. Third, longitudinal studies on cognition require repeated administration of cognitive tests, especially in a rather short period of time, which might lead to practice effects and improvement or maintenance of test scores despite a cognitive drop (36, 37). Although we adjusted the analysis for one potential type of practice effect by accounting for the change between the baseline and the first follow-up (which was also the largest change in cognitive scores for different tests), the increase over all cognitive tests remained. Thus, our study suggests an increase in cognitive performance over time in AF patients as the result of a practice effect, especially considering that the patients in the current cohort repeated the tests multiple times within a relatively short interval.

While practice effects are typically considered as biases when interpreting longitudinal studies on cognition, their use as markers of cognitive performance has gained interest. A recent systematic review on 27 studies on practice effect as cognitive marker indicated that smaller practice effects were associated with neurodegeneration biomarkers and thus might act as a potential marker of cognitive decline (38). Although our results showed a longitudinal increase in all cognitive measures due to practice effects, they indicate smaller increases in executive functioning, processing speed and general cognitive performance over time in patients with non-paroxysmal AF compared to patients with paroxysmal AF. Additionally, our results indicated no difference between AF groups in accumulating cases of cognitive drop defined as a threshold of >1 SD in all cognitive measures. When we altered the threshold value to >1.5 SD in the sensitivity analysis, a decrease in the number of events per visit was visible, but no difference in the results compared to the main analysis was found. As thresholds are set as a decrease from the first measurement, the fact that a person with a high score on the first measurement has more room for decrease than a person who scored low at baseline is not accounted for; similarly, using a threshold as an outcome does not account for possible practice effects. Furthermore, thresholds can be biased through temporary worse test results due to i.e., lack of motivation or wellbeing on testing day. Consequently, comparing practice effects, taken as the ability to learn over time, might act as a more reliable marker of cognitive change in longitudinal studies. Since our results indicate smaller practice effects in executive functioning, processing speed and general cognitive functioning, they might reflect reduced cognitive functioning in the non-paroxysmal AF group.

This study is among the first to investigate the differences in cognitive functions between non-paroxysmal and paroxysmal AF over time. One recent study investigated group differences between 90 persistent, 90 paroxysmal AF patients and 90 healthy participants using cognitive tests on memory, language, and visuospatial functions. While both AF groups showed lower cognitive performance compared to the healthy group, persistent and paroxysmal AF patients showed no differences on a total score of cognitive functioning, but a tendency towards smaller visuospatial abilities in the persistent AF group (39). Although no interaction between time and AF groups was found and permanent AF was not investigated by the authors, our results support the notion of lower cognitive abilities in the more sustained form of AF, which was categorized as non-paroxysmal AF in our study. Notably, our results indicated less practice effects in tasks addressing processing speed and executive functioning, which is in line with a previous study indicating an association between AF and a greater drop in executive functioning and processing speed (11). In extension to those findings, our results indicate that this association might be only present in patients with non-paroxysmal AF. Additional studies are needed to understand cognitive performance in the different subgroups more in detail, for example by addressing longitudinal change in different classifications of AF subtypes. Further, longer follow-up is required to gauge the full impact of AF type on cognitive decline.

Next to executive functioning and processing speed, the difference between AF subgroups was also present in the cognitive construct (CoCo) score (19). It has previously been shown that the CoCo score is likely to be more granular and more sensitive to detect small changes in cognitive function (19) which may be missed when examining each neurocognitive test alone (12, 40). Thus, using derived measures on cognitive functioning as well as tests on executive functioning and processing speed might be most sensitive in detecting subtle changes in cognition in AF patients, even after considering practice effects.

Shared risk factors of AF and cognitive decline are an important possible mechanism linking AF to alterations in cognitive function. In our study, patients with pre-existing depression, history of stroke or TIA and diabetes performed worse on all cognitive tests. Nonetheless, the impact of AF-type on longitudinal cognition was present in multivariate models suggesting some genuine effect of AF-type on cognition. Although AF and cognitive drop are likely to share one or more underlying pathogenic mechanisms, their possible pathophysiological aspects are still not fully understood (1, 26). Therefore, future studies with a control group are needed to explore the interplay between AF-type and other cardiovascular risk factors and comorbidities in relation to cognitive drop over time.

The large sample size of a comprehensively characterized, well-treated and representative cohort AF patients recruited from the main language regions of Switzerland, is a major strength of our study. Furthermore, brief composite measures (e.g., Mini-Mental State Examination) are commonly used as outcomes to assess cognitive performance, as they are time-efficient. Nevertheless, their assessment of cognitive functioning is limited by the insensitivity to detect subtle changes in various cognitive domains (12). Thus, the standardized assessment of cognitive functions using five validated and widely used cognitive measures in addition to the derived CoCo score (19) in our study displays a further strength, as it allows to study attention, psychomotor speed, and mental flexibility (executive control) as well as short-term memory, language, and visuospatial abilities. In addition, extensive information of AF-type, cardiac and neurological comorbidities were available and definitions of paroxysmal and non-paroxysmal AF were based on AF guidelines published in 2010 (20). Appropriate CRFs, which have been previously validated, were used (17, 18). Finally, all analyses were adjusted for potential confounders, and multiple sensitivity analyses provided consistent findings, supporting the robustness of the main findings.

Some limitations must be taken into account. First, understanding the role of AF itself in the changes in cognitive functioning would require the comparison to people without AF. We did not find a matching cohort of patients with no AF to perform such a comparison. The Swiss-AF control cohort is currently being recruited with the aim to make this analysis possible in the near future. Second, we identified 57 cases where MoCA and SF tests were not performed in the patient's mother tongue, which is a limitation to the results of these language dependent tests. Possibly more cases occurred, but it was not possible to precisely trace which cognitive tests were not performed in patients’ mother tongue, since this information could only be extracted from notes within the database by the study personnel. Nevertheless, the identified number of such cases is small, and dropping these patients led to a negligible change in the results. Third, missing data occurred in our sample, especially at follow-up 4 due to the COVID-19 pandemic that started during data collection and thus most cognitive tests could not be collected by phone (i.e., TMT-A, TMT-B, and DSST). Since the MoCA test conducted by telephone does not reflect the in-person test, we discarded those results. Participants were not included in the analyses if data was missing at baseline. Finally, although we adjusted our models to largest practice effects, identified primarily between the baseline and subsequent test measurements, the current study design did not allow for further statistical control for practice effects since time of follow-up and testing coincide almost completely. Nevertheless, our study supports previous notions that the lack of practice effect indicates a decline in cognitive performance over time. Future studies might further establish the role of practice effects as markers of cognitive decline by designing the study in a way that cognitive testing and follow-up are not at the same time point to control for the practice effect in subsequent measurements.

## 5. Conclusions

We found a small, constant increase in cognitive functioning over a median duration of 3.97 years in AF patients, which can probably be attributed to practice effects. While these results might indicate persistent learning abilities and maintenance of cognitive functions in patients with AF, smaller practice effects in the DSST, TMT-B and CoCo score as detected in patients with non-paroxysmal AF, might represent a potential early marker of later cognitive decline. Our study further highlights the importance of addressing comorbidities in AF early, as they were associated with worse cognitive performance. Therefore, future research should contribute to the understanding of underlying mechanisms in the relationship between AF and cognitive functioning.

## Data Availability

The datasets presented in this article are not readily available because of restrictions by the Ethics Committee. Requests to access the datasets should be directed to the corresponding author.

## References

[1] Puccio D Vizzini MC Baiamonte V Lunetta M Evola S Galassi AR Atrial fibrillation and cognitive disorders: an overview on possible correlation. Mech Ageing Dev. (2020) 191:111326. 10.1016/j.mad.2020.111326 32768444

[2] Kirchhof P Benussi S Kotecha D Ahlsson A Atar D Casadei B 2016 ESC guidelines for the management of atrial fibrillation developed in collaboration with EACTS. Eur J Cardiothorac Surg. (2016) 50:E1–E88. 10.1093/ejcts/ezw313 27663299

[3] Krijthe BP Kunst A Benjamin EJ Lip GY Franco OH Hofman A Projections on the number of individuals with atrial fibrillation in the European union, from 2000 to 2060. Eur Heart J. (2013) 34:2746–51. 10.1093/eurheartj/eht280 23900699 PMC3858024

[4] Wolf PA D'Agostino RB Belanger AJ Kannel WB. Probability of stroke: a risk profile from the framingham study. Stroke. (1991) 22:312–8. 10.1161/01.str.22.3.312 2003301

[5] Wang TJ Larson MG Levy D Vasan RS Leip EP Wolf PA Temporal relations of atrial fibrillation and congestive heart failure and their joint influence on mortality. Circulation. (2003) 107:2920–5. 10.1161/01.CIR.0000072767.89944.6E 12771006

[6] Ntaios G Sagris D Buckley BJR Harrison SL Abdul-Rahim A Austin P Risk of myocardial infarction and ischemic stroke in individuals with first-diagnosed paroxysmal vs. non-paroxysmal atrial fibrillation under anticoagulation. Europace. (2023) 25(6):euad143. 10.1093/europace/euad143 37285483 PMC10246817

[7] Benjamin EJ Wolf PA D'Agostino RB Silbershatz H Kannel WB Levy D. Impact of atrial fibrillation on the risk of death. Circulation. (1998) 98:946–52. 10.1161/01.cir.98.10.946 9737513

[8] Chen LY Sotoodehnia N Bůžková P Lopez FL Yee LM Heckbert SR Atrial fibrillation and the risk of sudden cardiac death: the atherosclerosis risk in communities study and cardiovascular health study. JAMA Intern Med. (2013) 173:29–35. 10.1001/2013.jamainternmed.744 23404043 PMC3578214

[9] Rivard L Friberg L Conen D Healey JS Berge T Boriani G Atrial fibrillation and dementia: a report from the AF-SCREEN international collaboration. Circulation. (2022) 145(5):392–409. 10.1161/CIRCULATIONAHA.121.055018. 35100023

[10] Koh YH Lew LZW Franke KB Elliott AD Lau DH Thiyagarajah A Predictive role of atrial fibrillation in cognitive decline: a systematic review and meta-analysis of 2.8 million individuals. Europace. (2022) 24(8):1229–39. 10.1093/europace/euac003 35061884 PMC9435641

[11] Chen LY Norby FL Gottesman RF Mosley TH Soliman EZ Agarwal SK Association of atrial fibrillation with cognitive decline and dementia over 20 years: the ARIC-NCS (atherosclerosis risk in communities neurocognitive study). J Am Heart Assoc. (2018) 7:e007301. 10.1161/JAHA.117.007301 29514809 PMC5907543

[12] Nishtala A Piers RJ Himali JJ Beiser AS Davis-Plourde KL Saczynski JS Atrial fibrillation and cognitive decline in the framingham heart study. Heart Rhythm. (2018) 15:166–72. 10.1016/j.hrthm.2017.09.036 28943482 PMC5881912

[13] de Bruijn RF Heeringa J Wolters FJ Franco OH Stricker BH Hofman A Association between atrial fibrillation and dementia in the general population. JAMA Neurol. (2015) 72:1288–94. 10.1001/jamaneurol.2015.2161 26389654

[14] Blum S Conen D. Mechanisms and clinical manifestations of cognitive decline in atrial fibrillation patients: potential implications for preventing dementia. Can J Cardiol. (2023) 39(2):159–71. 10.1016/j.cjca.2022.10.013 36252904

[15] Chiang CE Naditch-Brûlé L Murin J Goethals M Inoue H O’Neill J Distribution and risk profile of paroxysmal, persistent, and permanent atrial fibrillation in routine clinical practice. Circ Arrhythm Electrophysiol. (2012) 5:632–9. 10.1161/CIRCEP.112.970749 22787011

[16] Kim D Yang PS Yu HT Kim TH Jang E Sung JH Risk of dementia in stroke-free patients diagnosed with atrial fibrillation: data from a population-based cohort. Eur Heart J. (2019) 40:2313–23. 10.1093/eurheartj/ehz386 31212315

[17] Conen D Rodondi N Müller A Beer J Auricchio A Ammann P Design of the Swiss atrial fibrillation cohort study (Swiss-AF): structural brain damage and cognitive decline among patients with atrial fibrillation. Swiss Med Wkly. (2017) 147:w14467. 10.4414/smw.2017.14467 28695548

[18] Conen D Rodondi N Müller A Beer JH Ammann P Moschovitis G Relationships of overt and silent brain lesions with cognitive function in patients with atrial fibrillation. J Am Coll Cardiol. (2019) 73:989–99. 10.1016/j.jacc.2018.12.039 30846109

[19] Springer A Monsch AU Dutilh G Coslovsky M Kievit RA Bonati LH A factor score reflecting cognitive functioning in patients from the Swiss atrial fibrillation cohort study (Swiss-AF). PLoS One. (2020) 15:e0240167. 10.1371/journal.pone.0240167 33035257 PMC7546506

[20] Camm AJ Kirchhof P Lip GYH Schotten U Savelieva I Ernst S Guidelines for the management of atrial fibrillation: the task force for the management of atrial fibrillation of the European society of cardiology (ESC). Eur Heart J. (2010) 31:2369–429. 10.1093/eurheartj/ehq278 20802247

[21] Bano A Rodondi N Beer JH Moschovitis G Kobza R Aeschbacher S Association of diabetes with atrial fibrillation phenotype and cardiac and neurological comorbidities: insights from the Swiss-AF study. J Am Heart Assoc. (2021) 10:e021800. 10.1161/JAHA.121.021800 34753292 PMC8751921

[22] Montreal Cognitive Assessment. MoCA: a brief screening tool for mild cognitive impairment. J Am Geriatr Soc. (2005) 53:695–9. 10.1111/j.1532-5415.2005.53221.x 15817019

[23] Army Individual Test Battery: Manual of Directions and Scoring. Washington D.C.: War Department Adjutant General’s Office (1944).

[24] Smith SR Servesco AM Edwards JW Rahban R Barazani S Nowinski LA Exploring the validity of the comprehensive trail making test. Clin Neuropsychol. (2008) 22:507–18. 10.1080/13854040701399269 17853128

[25] Morris JC Heyman A Mohs RC Rogers H. The consortium to establish a registry for Alzheimer’s disease (CERAD). Part I. Clinical and neuropsychological assesment of Alzheimer’s disease. Neurology. (1989) 39:1159–65. 10.1212/wnl.39.9.1159 2771064

[26] Wechsler D. Wechsler adult intelligence scale–revised manual. New York: Psychological Corporation (1981).

[27] Matarazzo JD Herman DO. Base rate data for the WAIS-R: test-retest stability and VIQ-PIQ differences. J Clin Neuropsychol. (1984) 6:351–66. 10.1080/01688638408401227 The following formatting styles are meant as a guide, as long as the full citation is complete and clear, Frontiers referencing style will be applied during typesetting. 6501578

[28] Palmer K Fratiglioni L Winblad B. What is mild cognitive impairment? Variations in definitions and evolution of nondemented persons with cognitive impairment. Acta Neurol Scand. (2003) 107:14–20. 10.1034/j.1600-0404.107.s179.2.x 12603245

[29] Yesavage JA Brink TL Rose TL Lum O Huang V Adey M Development and validation of a geriatric depression screening scale: a preliminary report. J Psychiatr Res. (1982) 17:37–49. 10.1016/0022-3956(82)90033-4 7183759

[30] Bartels C Wegrzyn M Wiedl A Ackermann V Ehrenreich H. Practice effects in healthy adults: a longitudinal study on frequent repetitive cognitive testing. BMC Neurosci. (2010) 11:1–12. 10.1186/1471-2202-11-118 PMC2955045 20846444

[31] Hart RG Pearce LA Aguilar MI. Meta-analysis: antithrombotic therapy to prevent stroke in patients who have nonvalvular atrial fibrillation. Ann Intern Med. (2007) 146(12):857–67. 10.7326/0003-4819-146-12-200706190-00007 17577005

[32] Hicks T Stewart F Eisinga A. NOACs versus warfarin for stroke prevention in patients with AF: a systematic review and meta-analysis. Open Heart. (2016) 3(1):e000279. 10.1136/openhrt-2015-000279 26848392 PMC4731839

[33] Pendlebury ST Rothwell PM. Prevalence, incidence, and factors associated with pre-stroke and post-stroke dementia: a systematic review and meta-analysis. Lancet Neurol. (2009) 8:1006–18. 10.1016/S1474-4422(09)70236-4 19782001

[34] Pendlebury ST Rothwell PM Oxford Vascular S. Incidence and prevalence of dementia associated with transient ischaemic attack and stroke: analysis of the population-based Oxford vascular study. Lancet Neurol. (2019) 18:248–58. 10.1016/S1474-4422(18)30442-3 30784556 PMC6390174

[35] Van Beijsterveldt CE van Boxtel MP Bosma H Houx PJ Buntinx F Jolles J. Predictors of attrition in a longitudinal cognitive aging study: the Maastricht aging study (MAAS). J Clin Epidemiol. (2002) 55:216–23. 10.1016/s0895-4356(01)00473-5 11864790

[36] Abner EL Dennis BC Mathews MJ Mendiondo MS Caban-Holt A Kryscio RJ Practice effects in a longitudinal, multi-center Alzheimer’s disease prevention clinical trial. Trials. (2012) 13:217. 10.1186/1745-6215-13-217 23171483 PMC3543284

[37] Salthouse TA. Influence of age on practice effects in longitudinal neurocognitive change. Neuropsychology. (2010) 24:563–72. 10.1037/a0019026 20804244 PMC2933088

[38] Jutten RJ Grandoit E Foldi NS Sikkes SA Jones RN Choi SE Lower practice effects as a marker of cognitive performance and dementia risk: a literature review. Alzheimers Dement (Amst). (2020) 12:e12055. 10.1002/dad2.12055 32671181 PMC7346865

[39] Gaita F Corsinovi L Anselmino M Raimondo C Pianelli M Toso E Prevalence of silent cerebral ischemia in paroxysmal and persistent atrial fibrillation and correlation with cognitive function. J Am Coll Cardiol. (2013) 62:1990–7. 10.1016/j.jacc.2013.05.074 23850917

[40] Gibbons LE Carle AC Mackin RS Harvey D Mukherjee S Insel P A composite score for executive functioning, validated in Alzheimer’s disease neuroimaging initiative (ADNI) participants with baseline mild cognitive impairment. Brain Imaging Behav. (2012) 6:517–27. 10.1007/s11682-012-9176-1 22644789 PMC3684181

