# Supplementary material for: Mid-term changes in cognitive functions in patients with atrial fibrillation: a longitudinal analysis of the Swiss-AF cohort
Source: Front Cardiovasc Med. 2023 Aug 2;10:1212587. doi: 10.3389/fcvm.2023.1212587 (PMC10433225; doi:10.3389/fcvm.2023.1212587)
Supplement: Supplementary file 1 [file Datasheet1.pdf]

12 Supplementary Data

List of Swiss-AF investigators

University Hospital Basel and Basel University: Stefanie Aeschbacher, Katalin Bhend, Steffen Blum, Leo Bonati, Désirée Carmine, David Conen, Ceylan Eken, Urs Fischer, Corinne Girroy, Elisa Hennings, Philipp Krisai, Michael Kühne, Nina Mäder, Christine Meyer-Zürn, Pascal Meyre, Andreas U. Monsch, Luke Mosher, Christian Müller, Stefan Osswald, Rebecca E. Paladini, Raffaele Peter, Adrian Schweigler, Anne Springer, Christian Sticherling, Thomas Szucs, Gian Völlmin.

Principal Investigator: Stefan Osswald; Local Principal Investigator: Michael Kühne

University Hospital Bern: Faculty: Drahomir Aujesky, Juerg Fuhrer, Laurent Roten, Simon Jung, Heinrich Mattle; Research fellows: Seraina Netzer, Luise Adam, Carole Elodie Aubert, Martin Feller, Axel Loewe, Elisavet Moutzouri, Claudio Schneider; Study nurses: Tanja Flückiger, Cindy Groen, Lukas Ehrsam, Sven Hellrigl, Alexandra Nuoffer, Damiana Rakovic, Nathalie Schwab, Rylana Wenger, Tu Hanh Zarrabi Saffari. Local Principal Investigators: Nicolas Rodondi, Tobias Reichlin

Stadtspital Triemli Zurich: Christopher Beynon, Roger Dillier, Michèle Deubelbeiss, Franz Eberli, Christine Franzini, Isabel Juchli, Claudia Liedtke, Samira Murugiah, Jacqueline Nadler, Thayze Obst, Jasmin Roth, Fiona Schlomowitsch, Xiaoye Schneider, Katrin Studerus, Noreen Tynan, Dominik Weishaupt. Local Principal Investigator: Andreas Müller

Kantonspital Baden: Corinne Friedli, Silke Kuest, Karin Scheuch, Denise Hischier, Nicole Bonetti, Alexandra Grau, Jonas Villinger, Eva Laube, Philipp Baumgartner, Mark Filipovic, Marcel Frick, Giulia Montrasio, Stefanie Leuenberger, Franziska Rutz. Local Principal Investigator: Jürg-Hans Beer

Cardiocentro Lugano: Angelo Auricchio, Adriana Anesini, Cristina Camporini, Maria Luce Caputo, Francois Regoli, Martina Ronchi. Local Principal Investigator: Giulio Conte

Kantonsspital St. Gallen: Roman Brenner, David Altmann, Karin Fink, Michaela Gemperle. Local Principal Investigator: Peter Ammann

Hôpital Cantonal Fribourg: Mathieu Firmann, Sandrine Foucras, Martine Rime. Local Principal Investigator: Daniel Hayoz

Luzerner Kantonsspital: Benjamin Berte, Kathrin Bühler, Virgina Justi, Frauke Kellner-Weldon, Melanie Koch, Brigitta Mehmann, Sonja Meier, Myriam Roth, Andrea Ruckli-Kaeppeli, Ian Russi, Kai Schmidt, Mabelle Young, Melanie Zbinden. Local Principal Investigator: Richard Kobza

Ente Ospedaliero Cantonale Lugano: Maria Luisa De Perna, Elia Rigamonti, Carlo Cereda, Alessandro Cianfoni, Jane Frangi-Kultalahti, Patrizia Assunta Mayer Melchiorre, Anica Pin, Tatiana Terrot, Luisa Vicari. Local Principal Investigator: Giorgio Moschovitis.

University Hospital Geneva: Georg Ehret, Hervé Gallet, Elise Guillermet, Francois Lazeyras, Karl-Olof Lovblad, Patrick Perret, Philippe Tavel, Cheryl Teres. Local Principal Investigator: Dipen Shah

University Hospital Lausanne: Nathalie Lauriers, Marie Méan, Sandrine Salzmann, Jürg Schläpfer. Local Principal Investigator: Alessandra Pia Porretta

Bürgerspital Solothurn: Andrea Grêt, Jan Novak, Sandra Vitelli. Local Principal Investigator: Frank-Peter Stephan

Ente Ospedaliero Cantonale Bellinzona: Jane Frangi-Kultalahti, Augusto Gallino, Luisa Vicari. Local Principal Investigator: Marcello Di Valentino

University of Zurich/University Hospital Zurich: Helena Aebersold, Fabienne Foster, Matthias Schwenkglenks.

Medical Image Analysis Center AG Basel: Marco Düring, Tim Sinnecker, Anna Altermatt, Michael Amann, Petra Huber, Manuel Hürbin, Esther Ruberte, Alain Thöni, Jens Würfel, Vanessa Zuber.

Clinical Trial Unit Basel: Michael Coslovsky (Head), Pia Neuschwander, Patrick Simon, Olivia Wunderlin

Schiller AG Baar: Ramun Schmid, Christian Baumann

Table S1. Description of the neurocognitive test battery and all 17 items included in the cognitive assessment in the Swiss-AF study. Test description and items are grouped by test (MoCA, Trail Making Test Part A and B (TMT-A, TMT-B), Semantic Fluency Test (SF), and Digit Symbol Substitution Test (DSST). Information on definition of scores and measurement properties is also provided. The table was adapted according to Springer et al. [19].

**Item No**	**MoCA Items (scoring according to Manual; www.mocatest.org)**<br>*The Test evaluates visuospatial and executive functions, confrontation naming, memory, attention, language and abstraction [22].*
1	MoCA-Trail Making Test with letters and numbers; scored as "completed" vs "not completed": [0, 1]
2	Copy Cube; scored as "completed" vs "not completed": [1, 0]
3	Clock Drawing; scored as to how many of the three features are correct: [0, 1, 2, 3]
4	Naming Animals; scored as to the number of animals correctly named: [0, 1, 2, 3]
5	Digit Span forward; scored as "completed" vs "not completed": [1, 0]
6	Digit Span backward; scored as "completed" vs "not completed": [1, 0]
7	Letter A; scored as "completed" if less than 2 errors occurred: [1, 0]
8	100–7 (Serial 7 Subtraction); scored as: 0 correct [0 points], 1 through 3 correct [1 point], 4 correct [2 points], 5 correct [3 points]; values range from: [0, 1, 2, 3]
9	Sentence Repetition; scored according to number of sentences repeated correctly: [0, 1, 2]
10	F-Words, i.e., naming as many words that begin with the letter F; the number of correct words beginning with the letter F given in one minute [0,. . .]<br>(scoring within the MoCA total = 11 or more points [1], ten or less [0])
11	Abstraction; scored as the number of correct similarities [0, 1, 2]
12	Delayed Recall; scored as the number of words correctly recalled [0, 1, 2, 3, 4, 5]
13	Orientation; scored as the number of correct answers: [0, 1, 2, 3, 4, 5]
	**Trail Making Test Part A, (TMT-A) Item**<br>*The test measures visual attention and psychomotor speed [23]. Internal consistency has been reported with Cronbach's alpha = .86 to .88 [39].*
14	Outcome: number of correct connections per second: [0,. . .]
	**Trail Making Test Part B, (TMT-B) Item**<br>*The test assesses speed, accuracy and mental flexibility (e.g., task switching) [23]. Internal consistency has been reported with Cronbach's alpha = .86 to .88 [39].*

15	Outcome: number of correct connections per second: [0,. . .]
	**Semantic Fluency, Animals (SF), Item**<br>*The test measures semantic fluency- a combination of semantic memory and executive functions, complementing phonemic fluency within the MoCA [30].*
16	Number of correct animal names given in one minute: [0,. . .]
	**Digit Symbol Substitution Test (DSST), Item**<br>*The test assesses information processing speed, visuomotor coordination and attention [25].*<br>*DSST high test retest reliability has been reported. This test has high test–retest reliability [40].*
17	Number of correct symbols filled out in 120 seconds: [0,. . .]

Note. Cognitive Construct (CoCo) derived from the total of 17 items comprised in the five validated neuropsychological tests. Internal consistency for the coco score has been reported with Cronbach's alpha of .84 [19].

Text S1. Description of the age-education standardized cognitive function score

The Swiss-AF baseline data were used for standardization. A linear regression model was fit to the observed baseline data, for each subsequent observation the linear predictor was calculated. Finally, Z-scores were calculated by dividing the linear predictor by the residual standard error of the model. This model assumes a linear association between age and cognitive functioning, that was shown to be correct.

The standardization is performed via the following steps:

1. we fit a linear regression model to the observed values at baseline
2. for each subsequent observation (i.e. follow-up measurements) we calculate the linear predictor based on the model
3. to standardize (= calculate a Z-score) we divide the linear predictor by the residual standard error of the model (as fit using baseline values).

For purposes of the modeling, and to obtain meaningful expected values, we use as reference values (where relevant) the mean variable values for age and education level (years) at baseline based on the full Swiss-AF population. Thus, for example, we calculate the age and education adjusted Z-score for DSST using the following formula:

$$Z_{TMTA_i} = \frac{DSST_i - \beta_{age} \times (Age.at.visit - mean.age.BL) - \beta_{education-level} \times (Edu.Yrs - mean.edu.yrs.BL)}{\hat{\sigma}}$$

where σ is the square-root of the residual variance from the linear model.

Table S2. Number (and percentage) of missing tests per visit

	MoCA	SF	TMT-A	TMT-B	TMT B/A	DSST	CoCo
Baseline	13 (0.49)	7 (0.26)	22 (0.82)	35 (1.31)	35 (1.31)	19 (0.71)	56 (2.10)
Follow-up 1	279 (10.44)	271 (10.14)	281 (10.52)	290 (10.85)	290 (10.85)	291 (10.89)	307 (11.49)
Follow-up 2	399 (14.93)	386 (14.45)	397 (14.86)	409 (15.31)	412 (15.42)	417 (15.61)	438 (16.39)
Follow-up 3	648 (24.25)	491 (18.38)	646 (24.18)	655 (24.51)	656 (24.55)	659 (24.66)	677 (25.34)
Follow-up 4	941 (35.22)	471 (17.63)	940 (35.18)	949 (35.52)	949 (35.52)	944 (35.33)	955 (35.74)

Note. Patients who dropped out of the cohort are excluded already. CoCo: Cognitive construct; DSST: Digit Symbol Substitution Test; MoCA: Montreal Cognitive Assessment; SF: Semantic Fluency Test, animals; TMT-A: Trail Making Test A; TMT-B: Trail Making Test B; TMT B/A: ratio of Trail Making Test B/ Trail Making Test A.

Table S3. Number and percentage of cognitive measurements completed by visit

	n cognitive tests performed															
	**0**		**1**		**2**		**3**		**4**		**5**		**6**		**7**	
Visit	n	%	n	%	n	%	n	%	n	%	n	%	n	%	n	%
Baseline	2	0.083	3	0.124	1	0.041	16	0.663	17	0.704	17	0.704	1	0.041	2,358	97.640
Follow-up 1	268	11.227	6	0.251	5	0.209	3	0.126	8	0.335	17	0.712	2	0.084	2,078	87.055
Follow-up 2	382	16.696	8	0.350	5	0.219	3	0.131	17	0.743	23	1.005	2	0.087	1,848	80.769
Follow-up 3	486	22.304	156	7.159	3	0.138	3	0.138	7	0.321	22	1.010	2	0.092	1,500	68.839
Follow-up 4	464	24.029	475	24.599	2	0.104	2	0.104	8	0.414	4	0.207	1	0.052	975	50.492

Table S4. Overview of the number of dropouts with reasons per visit

	Baseline	Follow-up 1	Follow-up 2	Follow-up 3
Patient cannot be reached	3	5	5	10
Consent withdrawal	25	26	21	27
Death	0	68	82	103
Loss to follow-up / FU4 visit late	0	0	0	109

Table S5. Estimates with 95% CI for MoCA. The results for each covariate represent the effect after adjusting for all other variables in the model

	estimate	95 % CI
AF-type (non-paroxysmal over paroxysmal)	-0.06	[-0.14, 0.01]
Time (years)	0.09	[0.06, 0.12]
First visit (yes over no)	-0.09	[-0.13, -0.05]
Sex (female over male)	0.20	[0.12, 0.28]
History of stroke or TIA (yes over no)	-0.18	[-0.27, -0.10]
History of diabetes (yes over no)	-0.31	[-0.41, -0.22]
Arterial hypertension (yes over no)	-0.08	[-0.16, -0.00]
Depression (yes over no)	-0.24	[-0.37, -0.11]
Oral anti-coagulant medication (yes over no)	0.07	[-0.05, 0.19]
Active smoker (yes over no)	-0.10	[-0.23, 0.04]
GFR	0.00	[-0.00, 0.00]
Age at baseline (years)	-0.00	[-0.01, 0.00]
AF-type (non-paroxysmal over paroxysmal):Time (years)	-0.01	[-0.04, 0.01]

Note. AF: Atrial fibrillation; CI: Confidence interval; GFR: Glomerular filtration rate; MoCA: Montreal Cognitive Assessment; TIA: Transient ischemic attack.

Table S6. Estimates with 95% CI for SF. The results for each covariate represent the effect after adjusting for all other variables in the model

	estimate	95 % CI
AF-type (non-paroxysmal over paroxysmal)	-0.03	[-0.10, 0.05]
Time (years)	0.03	[0.00, 0.05]
First visit (yes over no)	-0.07	[-0.12, -0.03]
Sex (female over male)	0.16	[0.08, 0.24]
History of stroke or TIA (yes over no)	-0.21	[-0.30, -0.12]
History of diabetes (yes over no)	-0.24	[-0.34, -0.15]
Arterial hypertension (yes over no)	-0.04	[-0.12, 0.04]
Depression (yes over no)	-0.24	[-0.37, -0.10]
Oral anti-coagulant medication (yes over no)	0.02	[-0.10, 0.14]
Active smoker (yes over no)	0.04	[-0.10, 0.18]
GFR	0.00	[-0.00, 0.00]
Age at baseline (years)	-0.00	[-0.01, 0.00]
AF-type (non-paroxysmal over paroxysmal):Time (years)	0.01	[-0.02, 0.03]

Note. AF: Atrial fibrillation; CI: Confidence interval; GFR: Glomerular filtration rate; SF: Semantic Fluency Test, animals; TIA: Transient ischemic attack.

Table S7. Estimates with 95% CI for TMT-A. The results for each covariate represent the effect after adjusting for all other variables in the model

	estimate	95 % CI
AF-type (non-paroxysmal over paroxysmal)	-0.06	[-0.14, 0.02]
Time (years)	0.09	[0.07, 0.12]
First visit (yes over no)	-0.11	[-0.16, -0.06]
Sex (female over male)	0.01	[-0.07, 0.09]
History of stroke or TIA (yes over no)	-0.23	[-0.32, -0.14]
History of diabetes (yes over no)	-0.20	[-0.30, -0.11]
Arterial hypertension (yes over no)	0.04	[-0.04, 0.12]
Depression (yes over no)	-0.28	[-0.42, -0.15]
Oral anti-coagulant medication (yes over no)	-0.09	[-0.22, 0.03]
Active smoker (yes over no)	-0.11	[-0.25, 0.03]
GFR	0.00	[0.00, 0.00]
Age at baseline (years)	-0.00	[-0.01, 0.00]
AF-type (non-paroxysmal over paroxysmal):Time (years)	-0.01	[-0.04, 0.01]

Note. AF: Atrial fibrillation; CI: Confidence interval; GFR: Glomerular filtration rate; TMT-A: Trail Making Test A; TIA: Transient ischemic attack.

Table S8. Estimates with 95% CI for TMT-B. The results for each covariate represent the effect after adjusting for all other variables in the model

	estimate	95 % CI
AF-type (non-paroxysmal over paroxysmal)	-0.03	[-0.11, 0.05]
Time (years)	0.10	[0.07, 0.13]
First visit (yes over no)	-0.06	[-0.11, -0.02]
Sex (female over male)	0.04	[-0.04, 0.13]
History of stroke or TIA (yes over no)	-0.22	[-0.32, -0.13]
History of diabetes (yes over no)	-0.32	[-0.42, -0.22]
Arterial hypertension (yes over no)	-0.03	[-0.11, 0.06]
Depression (yes over no)	-0.37	[-0.51, -0.24]
Oral anti-coagulant medication (yes over no)	-0.12	[-0.24, 0.01]
Active smoker (yes over no)	-0.15	[-0.29, -0.00]
GFR	0.00	[-0.00, 0.00]
Age at baseline (years)	-0.00	[-0.01, 0.00]
AF-type (non-paroxysmal over paroxysmal):Time (years)	-0.03	[-0.05, -0.00]

Note. AF: Atrial fibrillation; CI: Confidence interval; GFR: Glomerular filtration rate; TMT-B: Trail Making Test B; TIA: Transient ischemic attack.

Table S9. Estimates with 95% CI for TMT-B / TMT-A. The results for each covariate represent the effect after adjusting for all other variables in the model

	estimate	95 % CI
AF-type (non-paroxysmal over paroxysmal)	0.00	[-0.07, 0.07]
Time (years)a	0.02	[-0.01, 0.05]
First visit (yes over no)	0.03	[-0.02, 0.08]
Sex (female over male)	0.04	[-0.03, 0.10]
History of stroke or TIA (yes over no)	-0.07	[-0.14, 0.01]
History of diabetes (yes over no)	-0.16	[-0.24, -0.08]
Arterial hypertension (yes over no)	-0.04	[-0.10, 0.03]
Depression (yes over no)	-0.13	[-0.24, -0.02]
Oral anti-coagulant medication (yes over no)	-0.03	[-0.13, 0.07]
Active smoker (yes over no)	-0.08	[-0.19, 0.03]
GFR	-0.00	[-0.00, 0.00]
Age at baseline (years)	-0.00	[-0.00, 0.00]
AF-type (non-paroxysmal over paroxysmal):Time (years)a	-0.01	[-0.04, 0.01]

Note. AF: Atrial fibrillation; CI: Confidence interval; GFR: Glomerular filtration rate; TMT B/A: ratio of Trail Making Test B/ Trail Making Test A; TIA: Transient ischemic attack.

Table S10. Estimates with 95% CI for DSST. The results for each covariate represent the effect after adjusting for all other variables in the model

	estimate	95 % CI
AF-type (non-paroxysmal over paroxysmal)	-0.07	[-0.15, 0.01]
Time (years)	0.07	[0.06, 0.08]
First visit (yes over no)	-0.04	[-0.07, -0.01]
Sex (female over male)	0.23	[0.14, 0.32]
History of stroke or TIA (yes over no)	-0.32	[-0.41, -0.22]
History of diabetes (yes over no)	-0.33	[-0.43, -0.22]
Arterial hypertension (yes over no)	-0.03	[-0.11, 0.06]
Depression (yes over no)	-0.29	[-0.43, -0.14]
Oral anti-coagulant medication (yes over no)	-0.07	[-0.20, 0.06]
Active smoker (yes over no)	-0.19	[-0.34, -0.04]
GFR	0.00	[0.00, 0.01]
Age at baseline (years)	-0.00	[-0.01, 0.01]
AF-type (non-paroxysmal over paroxysmal):Time (years)	-0.02	[-0.04, -0.00]

Note. AF: Atrial fibrillation; CI: Confidence interval; DSST: Digit Symbol Substitution Test; GFR: Glomerular filtration rate; TIA: Transient ischemic attack.

Table S11. Estimates with 95% CI for CoCo. The results for each covariate represent the effect after adjusting for all other variables in the model

	estimate	95 % CI
AF-type (non-paroxysmal over paroxysmal)	-0.05	[-0.13, 0.03]
Time (years)	0.11	[0.09, 0.13]
First visit (yes over no)	0.05	[0.02, 0.08]
Sex (female over male)	0.14	[0.05, 0.23]
History of stroke or TIA (yes over no)	-0.29	[-0.39, -0.19]
History of diabetes (yes over no)	-0.40	[-0.51, -0.30]
Arterial hypertension (yes over no)	-0.04	[-0.13, 0.05]
Depression (yes over no)	-0.37	[-0.51, -0.22]
Oral anti-coagulant medication (yes over no)	-0.07	[-0.20, 0.06]
Active smoker (yes over no)	-0.14	[-0.29, 0.01]
GFR	0.00	[0.00, 0.01]
Age at baseline (years)	0.00	[-0.00, 0.01]
AF-type (non-paroxysmal over paroxysmal):Time (years)	-0.03	[-0.04, -0.01]

Note. AF: Atrial fibrillation; CI: Confidence interval; CoCo: Cognitive construct; GFR: Glomerular filtration rate; TIA: Transient ischemic attack.

Table S12. Number (percentages) of new cases of cognitive drop per visit. Patients with a drop are excluded from analyses of later timepoints

	MoCA	SF	TMT-A	TMT-B	TMT B/A	DSST	CoCo
Follow-up 1	104 (3.9)	181 (6.8)	184 (6.9)	153 (5.7)	316 (11.8)	89 (3.3)	112 (4.2)
Follow-up 2	69 (2.6)	117 (4.4)	100 (3.7)	80 (3.0)	152 (5.7)	49 (1.8)	24 (0.9)
Follow-up 3	34 (1.3)	97 (3.6)	50 (1.9)	51 (1.9)	77 (2.9)	24 (0.9)	32 (1.2)
Follow-up 4	21 (0.8)	77 (2.9)	18 (0.7)	26 (1.0)	46 (1.7)	22 (0.8)	22 (0.8)

Note: Data are presented as counts (percentages). CoCo: Cognitive construct; DSST: Digit Symbol Substitution Test; MoCA: Montreal Cognitive Assessment; SF: Semantic Fluency Test, animals; TMT-A: Trail Making Test A; TMT-B: Trail Making Test B; TMT B/A: ratio of Trail Making Test B/ Trail Making Test A.

Table S13. HR with 95% CI for MoCA impairment. The results for each covariate represent the effect after adjusting for all other variables in the model

	HR	95 % CI
AF-type (non-paroxysmal over paroxysmal)	1.04	[0.80, 1.36]
Sex (female over male)	0.99	[0.73, 1.33]
History of stroke or TIA (yes over no)	1.43	[1.06, 1.93]
History of diabetes (yes over no)	1.39	[0.98, 1.96]
Arterial hypertension (yes over no)	0.97	[0.73, 1.31]
Depression (yes over no)	1.26	[0.79, 2.00]
Oral anti-coagulant medication (yes over no)	0.95	[0.60, 1.51]
Active smoker (yes over no)	1.55	[0.94, 2.53]
Age at baseline (years)	1.07	[1.05, 1.09]

Note. AF: Atrial fibrillation; CI: Confidence interval; MoCA: Montreal Cognitive Assessment; TIA: Transient ischemic attack.

Table S14. HR with 95% CI for SF impairment. The results for each covariate represent the effect after adjusting for all other variables in the model

	HR	95 % CI
AF-type (non-paroxysmal over paroxysmal)	1.01	[0.83, 1.22]
Sex (female over male)	0.87	[0.70, 1.08]
History of stroke or TIA (yes over no)	1.24	[0.99, 1.54]
History of diabetes (yes over no)	1.12	[0.86, 1.44]
Arterial hypertension (yes over no)	1.07	[0.87, 1.31]
Depression (yes over no)	1.00	[0.69, 1.44]
Oral anti-coagulant medication (yes over no)	1.19	[0.85, 1.66]
Active smoker (yes over no)	1.13	[0.78, 1.64]
Age at baseline (years)	1.02	[1.00, 1.03]

Note. AF: Atrial fibrillation; CI: Confidence interval; SF: Semantic Fluency Test, animals; TIA: Transient ischemic attack.

Table S15. HR with 95% CI for TMT-A impairment. The results for each covariate represent the effect after adjusting for all other variables in the model

	HR	95 % CI
AF-type (non-paroxysmal over paroxysmal)	1.15	[0.92, 1.43]
Sex (female over male)	0.99	[0.77, 1.26]
History of stroke or TIA (yes over no)	0.98	[0.74, 1.29]
History of diabetes (yes over no)	1.33	[1.01, 1.76]
Arterial hypertension (yes over no)	1.15	[0.90, 1.46]
Depression (yes over no)	0.84	[0.53, 1.34]
Oral anti-coagulant medication (yes over no)	1.19	[0.81, 1.76]
Active smoker (yes over no)	0.92	[0.60, 1.41]
Age at baseline (years)	0.99	[0.98, 1.01]

Note. AF: Atrial fibrillation; CI: Confidence interval; TMT-A: Trail Making Test A; TIA: Transient ischemic attack.

Table S16 HR with 95% CI for TMT-B impairment. The results for each covariate represent the effect after adjusting for all other variables in the model

	HR	95 % CI
AF-type (non-paroxysmal over paroxysmal)	0.94	[0.75, 1.19]
Sex (female over male)	1.02	[0.79, 1.32]
History of stroke or TIA (yes over no)	1.25	[0.95, 1.66]
History of diabetes (yes over no)	0.61	[0.41, 0.89]
Arterial hypertension (yes over no)	1.00	[0.78, 1.27]
Depression (yes over no)	0.84	[0.54, 1.32]
Oral anti-coagulant medication (yes over no)	1.04	[0.71, 1.52]
Active smoker (yes over no)	1.11	[0.72, 1.72]
Age at baseline (years)	1.01	[1.00, 1.03]

Note. AF: Atrial fibrillation; CI: Confidence interval; TMT-B: Trail Making Test B; TIA: Transient ischemic attack.

Table S17. HR with 95% CI for TMT-B/TMT-A impairment. The results for each covariate represent the effect after adjusting for all other variables in the model

	HR	95 % CI
AF-type (non-paroxysmal over paroxysmal)	0.98	[0.83, 1.16]
Sex (female over male)	1.02	[0.84, 1.22]
History of stroke or TIA (yes over no)	1.16	[0.95, 1.42]
History of diabetes (yes over no)	0.82	[0.64, 1.05]
Arterial hypertension (yes over no)	0.99	[0.83, 1.19]
Depression (yes over no)	0.78	[0.55, 1.12]
Oral anti-coagulant medication (yes over no)	0.90	[0.69, 1.19]
Active smoker (yes over no)	1.45	[1.06, 1.98]
Age at baseline (years)	1.03	[1.02, 1.04]

Note. AF: Atrial fibrillation; CI: Confidence interval; TMT B/A: ratio of Trail Making Test B/ Trail Making Test A; TIA: Transient ischemic attack.

Table S18. HR with 95% CI for DSST impairment. The results for each covariate represent the effect after adjusting for all other variables in the model

	HR	95 % CI
AF-type (non-paroxysmal over paroxysmal)	0.98	[0.72, 1.32]
Sex (female over male)	0.97	[0.69, 1.36]
History of stroke or TIA (yes over no)	1.46	[1.04, 2.04]
History of diabetes (yes over no)	1.66	[1.15, 2.40]
Arterial hypertension (yes over no)	0.86	[0.62, 1.18]
Depression (yes over no)	1.05	[0.59, 1.88]
Oral anti-coagulant medication (yes over no)	1.01	[0.61, 1.69]
Active smoker (yes over no)	1.58	[0.94, 2.66]
Age at baseline (years)	1.04	[1.01, 1.06]

Note. AF: Atrial fibrillation; CI: Confidence interval; DSST: Digit Symbol Substitution Test; TIA: Transient ischemic attack.

Table S19. HR with 95% CI for CoCo impairment. The results for each covariate represent the effect after adjusting for all other variables in the model

	HR	95 % CI
AF-type (non-paroxysmal over paroxysmal)	0.90	[0.67, 1.20]
Sex (female over male)	0.70	[0.49, 1.00]
History of stroke or TIA (yes over no)	1.47	[1.05, 2.05]
History of diabetes (yes over no)	1.06	[0.71, 1.59]
Arterial hypertension (yes over no)	0.92	[0.67, 1.26]
Depression (yes over no)	1.34	[0.79, 2.28]
Oral anti-coagulant medication (yes over no)	0.86	[0.53, 1.40]
Active smoker (yes over no)	1.62	[0.95, 2.77]
Age at baseline (years)	1.04	[1.02, 1.06]

Note. AF: Atrial fibrillation; CI: Confidence interval; CoCo: Cognitive construct; TIA: Transient ischemic attack.

Table S20. Baseline characteristics between the "worst" group (i.e. dropout) and the patients with a complete data set

	Full (all 5 visits)	Worst (= drop out)
n	1475	940
Age at baseline (y) (mean (SD))	71.75 (8.30)	75.57 (8.10)
Sex = Female (%)	378 (25.6)	284 (30.2)
Highest education level (%)		
Basic	151 (10.2)	137 (14.6)
Middle	708 (48.0)	489 (52.2)
Advanced	615 (41.7)	311 (33.2)
Years of education (mean (SD))	13.28 (3.24)	12.39 (3.13)
History of stroke/TIA = Yes (%)	257 (17.4)	223 (23.8)
History of diabetes = Yes (%)	193 (13.1)	229 (24.4)
History of hypertension = Yes (%)	986 (66.8)	705 (75.0)
History of depression = Yes (%)	94 (6.4)	106 (11.3)
Antocoagulant medication = Yes (%)	1325 (89.8)	857 (91.2)
GFR (mean (SD))	62.19 (17.05)	54.48 (19.18)
Active smoker = Yes (%)	92 (6.2)	83 (8.8)

Data are presented as mean (± SD) or counts (percentages). GFR: glomerular filtration rate; min.: minutes; ml: milliliter; TIA: transient ischemic attack; *Basic education: ≤6 years (less than compulsory education curriculum); middle education: 6 to ≤12 years (high school or similar); advanced education: ≥12 years (college or university degree).

Table S21. Results of three-way interactions with time and AF-type

Interaction term	Outcome	P-value
sex	MoCA	0.658
	SF	0.502
	TMT-A	0.558
	TMT-B	0.139
	TMT-A/B	0.641
	DSST	0.890
	CoCo	0.198
stroke	MoCA	0.502
	SF	0.412
	TMT-A	0.882
	TMT-B	0.690
	TMT-A/B	0.987
	DSST	0.502
	CoCo	0.990
smoking	MoCA	0.253
	SF	0.924
	TMT-A	0.360
	TMT-B	0.249
	TMT-A/B	0.426
	DSST	0.643
	CoCo	0.230
diabetes	MoCA	0.180
	SF	0.609
	TMT-A	0.931
	TMT-B	0.985
	TMT-A/B	0.405
	DSST	0.022
	CoCo	0.486
hypertension	MoCA	0.006
	SF	0.918
	TMT-A	0.432
	TMT-B	0.040
	TMT-A/B	0.307
	DSST	0.014
	CoCo	0.013
depression	MoCA	0.318
	SF	0.987
	TMT-A	0.619
	TMT-B	0.382
	TMT-A/B	0.259
	DSST	0.149
	CoCo	0.293

Note. CoCo: Cognitive construct; DSST: Digit Symbol Substitution Test; MoCA: Montreal Cognitive Assessment; SF: Semantic Fluency Test, animals; TMT-A: Trail Making Test A; TMT-B: Trail Making Test B; TMT B/A: ratio of Trail Making Test B/ Trail Making Test A.

Table S22. Estimate with 95% CI for MoCA in patients with (left) and without (right) a history of hypertension

	Hypertension		No Hypertension	
Variable	estimate	95 % CI	estimate	95 % CI
AF-type (non-paroxysmal over paroxysmal)	-0.04	[-0.13, 0.05]	-0.02	[-0.15, 0.11]
Time (years)	0.09	[0.07, 0.12]	0.13	[0.10, 0.16]
First visit (yes over no)	-0.09	[-0.13, -0.04]	-0.10	[-0.17, -0.04]
Sex (female over male)	0.21	[0.11, 0.31]	0.22	[0.08, 0.35]
History of stroke or TIA (yes over no)	-0.14	[-0.25, -0.03]	-0.26	[-0.42, -0.11]
History of diabetes (yes over no)	-0.39	[-0.50, -0.28]	-0.13	[-0.37, 0.11]
Depression (yes over no)	-0.19	[-0.36, -0.03]	-0.24	[-0.47, -0.00]
Oral anti-coagulant medication (yes over no)	0.18	[0.02, 0.35]	-0.03	[-0.21, 0.14]
Active smoker (yes over no)	-0.08	[-0.26, 0.09]	-0.22	[-0.47, 0.02]
GFR	0.00	[-0.00, 0.00]	0.00	[-0.00, 0.01]
Age at baseline (years)	-0.01	[-0.01, 0.00]	0.00	[-0.00, 0.01]
AF-type (non-paroxysmal over paroxysmal):Time (years)	0.00	[-0.03, 0.03]	-0.06	[-0.10, -0.03]

Note. AF: Atrial fibrillation; CI: Confidence interval; GFR: Glomerular filtration rate; MoCA: Montreal Cognitive Assessment; TIA: Transient ischemic attack.

Table S23. Estimate with 95% CI for TMT-B in patients with (left) and without (right) a history of hypertension

	Hypertension		No Hypertension	
Variable	estimate	95 % CI	estimate	95 % CI
AF-type (non-paroxysmal over paroxysmal)	-0.04	[-0.13, 0.05]	0.03	[-0.11, 0.18]
Time (years)	0.08	[0.05, 0.10]	0.13	[0.09, 0.17]
First visit (yes over no)	-0.07	[-0.12, -0.02]	-0.05	[-0.13, 0.03]
Sex (female over male)	0.07	[-0.03, 0.17]	-0.03	[-0.19, 0.12]
History of stroke or TIA (yes over no)	-0.21	[-0.32, -0.10]	-0.22	[-0.40, -0.04]
History of diabetes (yes over no)	-0.33	[-0.44, -0.22]	-0.31	[-0.59, -0.03]
Depression (yes over no)	-0.33	[-0.49, -0.17]	-0.51	[-0.78, -0.25]
Oral anti-coagulant medication (yes over no)	0.13	[-0.04, 0.29]	-0.53	[-0.73, -0.33]
Active smoker (yes over no)	-0.10	[-0.27, 0.07]	-0.32	[-0.60, -0.04]
GFR	0.00	[-0.00, 0.00]	-0.00	[-0.01, 0.00]
Age at baseline (years)	-0.00	[-0.01, 0.00]	-0.01	[-0.02, -0.00]
AF-type (non-paroxysmal over paroxysmal):Time (years)	-0.01	[-0.04, 0.02]	-0.06	[-0.11, -0.02]

Note. AF: Atrial fibrillation; CI: Confidence interval; GFR: Glomerular filtration rate; TMT-B: Trail Making Test B; TIA: Transient ischemic attack.

Table S24. Estimate with 95% CI for DSST in patients with (left) and without (right) a history of hypertension

	Hypertension		No Hypertension	
Variable	estimate	95 % CI	estimate	95 % CI
AF-type (non-paroxysmal over paroxysmal)	-0.08	[-0.17, 0.02]	-0.06	[-0.20, 0.08]
Time (years)	0.05	[0.03, 0.07]	0.10	[0.08, 0.12]
First visit (yes over no)	-0.04	[-0.08, -0.00]	-0.03	[-0.09, 0.02]
Sex (female over male)	0.25	[0.14, 0.35]	0.21	[0.05, 0.37]
History of stroke or TIA (yes over no)	-0.32	[-0.43, -0.20]	-0.30	[-0.48, -0.12]
History of diabetes (yes over no)	-0.34	[-0.45, -0.22]	-0.30	[-0.58, -0.01]
Depression (yes over no)	-0.25	[-0.42, -0.09]	-0.37	[-0.64, -0.10]
Oral anti-coagulant medication (yes over no)	0.18	[0.01, 0.35]	-0.42	[-0.62, -0.22]
Active smoker (yes over no)	-0.13	[-0.30, 0.05]	-0.39	[-0.68, -0.11]
GFR	0.00	[0.00, 0.01]	0.00	[-0.00, 0.01]
Age at baseline (years)	0.00	[-0.01, 0.01]	-0.00	[-0.01, 0.01]
AF-type (non-paroxysmal over paroxysmal):Time (years)	-0.00	[-0.02, 0.02]	-0.05	[-0.08, -0.02]

Note. AF: Atrial fibrillation; CI: Confidence interval; DSST: Digit Symbol Substitution Test; GFR: Glomerular filtration rate; TIA: Transient ischemic attack.

Table S25. Estimate with 95% CI for CoCo in patients with (left) and without (right) a history of hypertension

	Hypertension		No Hypertension	
Variable	estimate	95 % CI	estimate	95 % CI
AF-type (non-paroxysmal over paroxysmal)	-0.05	[-0.15, 0.05]	-0.03	[-0.17, 0.11]
Time (years)	0.09	[0.07, 0.10]	0.13	[0.11, 0.16]
First visit (yes over no)	0.05	[0.01, 0.09]	0.05	[-0.01, 0.10]
Sex (female over male)	0.17	[0.06, 0.28]	0.10	[-0.05, 0.26]
History of stroke or TIA (yes over no)	-0.28	[-0.39, -0.16]	-0.30	[-0.48, -0.12]
History of diabetes (yes over no)	-0.43	[-0.54, -0.31]	-0.40	[-0.68, -0.12]
Depression (yes over no)	-0.31	[-0.47, -0.14]	-0.52	[-0.79, -0.26]
Oral anti-coagulant medication (yes over no)	0.20	[0.03, 0.37]	-0.47	[-0.67, -0.26]
Active smoker (yes over no)	-0.07	[-0.25, 0.11]	-0.34	[-0.63, -0.06]
GFR	0.00	[0.00, 0.01]	-0.00	[-0.00, 0.00]
Age at baseline (years)	0.00	[-0.01, 0.01]	-0.00	[-0.01, 0.01]
AF-type (non-paroxysmal over paroxysmal):Time (years)	-0.01	[-0.03, 0.01]	-0.06	[-0.09, -0.03]

Note. AF: Atrial fibrillation; CI: Confidence interval; CoCo: Cognitive construct; GFR: Glomerular filtration rate; TIA: Transient ischemic attack.

Table S26. Number (and percentage) of new cases of cognitive drop per visit with the impairment threshold at 1.5 SD

	MoCA	SF	TMT-A	TMT-B	TMT B/A	DSST	CoCo
Follow-up 1	49 (1.83)	86 (3.22)	70 (2.62)	63 (2.36)	155 (5.80)	27 (1.01)	26 (0.97)
Follow-up 2	37 (1.38)	67 (2.51)	41 (1.53)	38 (1.42)	77 (2.88)	23 (0.86)	6 (0.22)
Follow-up 3	21 (0.79)	38 (1.42)	21 (0.79)	22 (0.82)	55 (2.06)	5 (0.19)	5 (0.19)
Follow-up 4	10 (0.37)	33 (1.24)	13 (0.49)	16 (0.60)	27 (1.01)	9 (0.34)	7 (0.26)

Note. CoCo: Cognitive construct; DSST: Digit Symbol Substitution Test; MoCA: Montreal Cognitive Assessment; SD: Standard deviation; SF: Semantic Fluency Test, animals; TMT-A: Trail Making Test A; TMT-B: Trail Making Test B; TMT B/A: ratio of Trail Making Test B/ Trail Making Test A.

Table S27. HR with 95% CI for SF impairment (cut-off 1.5 SD)

	HR	95 % CI
AF-type (non-paroxysmal over paroxysmal)	0.88	[0.67, 1.15]
Sex (female over male)	0.69	[0.49, 0.96]
History of stroke or TIA (yes over no)	1.12	[0.81, 1.56]
History of diabetes (yes over no)	1.17	[0.81, 1.68]
Arterial hypertension (yes over no)	1.01	[0.75, 1.36]
Depression (yes over no)	1.37	[0.84, 2.23]
Oral anti-coagulant medication (yes over no)	1.46	[0.87, 2.46]
Active smoker (yes over no)	1.03	[0.60, 1.77]
Age at baseline (years)	1.01	[0.99, 1.02]

Note. AF: Atrial fibrillation; CI: Confidence interval; SD: Standard deviation; SF: Semantic Fluency Test, animals; TIA: Transient ischemic attack.

Table S28. Estimates with 95% CI for the linear mixed effects model for SF including patients who did not miss any visit. The results for each covariate represent the effect after adjusting for all other variables in the model

	estimate	95 % CI
AF-type (non-paroxysmal over paroxysmal)	-0.00	[-0.10, 0.10]
Time (years)	0.04	[0.01, 0.06]
First visit (yes over no)	-0.10	[-0.15, -0.04]
Sex (female over male)	0.20	[0.10, 0.31]
History of stroke or TIA (yes over no)	-0.10	[-0.22, -0.02]
History of diabetes (yes over no)	-0.24	[-0.38, -0.11]
Arterial hypertension (yes over no)	-0.02	[-0.12, 0.08]
Depression (yes over no)	-0.41	[-0.59, -0.22]
Oral anti-coagulant medication (yes over no)	-0.10	[-0.25, 0.05]
Active smoker (yes over no)	0.19	[-0.00, 0.38]
GFR (milliliter/minute)	0.00	[-0.03, 0.03]
Age at baseline (10 years)	-0.02	[-0.08, 0.04]
AF-type (non-paroxysmal over paroxysmal):Time (years)	0.00	[-0.02, 0.03]

Note. AF: Atrial fibrillation; CI: Confidence interval; GFR: Glomerular filtration rate; SF: Semantic Fluency Test, animals; TIA: Transient ischemic attack.

Table S29. Estimates with 95% CI for the linear mixed effects model for SF in the group of patients that dropped out. The results for each covariate represent the effect after adjusting for all other variables in the model

	estimate	95 % CI
AF-type (non-paroxysmal over paroxysmal)	-0.07	[-0.19, 0.05]
Time (years)	-0.00	[-0.05, 0.04]
First visit (yes over no)	-0.03	[-0.11, 0.06]
Sex (female over male)	0.10	[-0.03, 0.22]
History of stroke or TIA (yes over no)	-0.26	[-0.40, -0.13]
History of diabetes (yes over no)	-0.19	[-0.33, -0.05]
Arterial hypertension (yes over no)	-0.04	[-0.17, 0.10]
Depression (yes over no)	-0.09	[-0.27, 0.10]
Oral anti-coagulant medication (yes over no)	0.20	[0.00, 0.40]
Active smoker (yes over no)	-0.13	[-0.33, 0.07]
GFR	0.00	[-0.00, 0.00]
AF-type (non-paroxysmal over paroxysmal):Time (years)	0.03	[-0.02, 0.09]

Note. AF: Atrial fibrillation; CI: Confidence interval; GFR: Glomerular filtration rate; SF: Semantic Fluency Test, animals; TIA: Transient ischemic attack.

Table S30. Estimates with 95% CI for SF in the model with inverse probability of censoring weights added. The results for each covariate represent the effect after adjusting for all other variables in the model

	estimate	95 % CI
AF-type (non-paroxysmal over paroxysmal)	-0.03	[-0.11, 0.04]
Time (years)	0.03	[0.01, 0.05]
First visit (yes over no)	-0.07	[-0.12, -0.03]
Sex (female over male)	0.15	[0.07, 0.23]
History of stroke or TIA (yes over no)	-0.18	[-0.27, -0.09]
History of diabetes (yes over no)	-0.25	[-0.34, -0.15]
Arterial hypertension (yes over no)	-0.04	[-0.12, -0.04]
Depression (yes over no)	-0.28	[-0.42, -0.15]
Oral anti-coagulant medication (yes over no)	0.01	[-0.12, 0.13]
Active smoker (yes over no)	0.02	[-0.12, 0.16]
GFR	0.00	[-0.00, 0.00]
Age at baseline (years)	-0.00	[-0.01, -0.00]
AF-type (non-paroxysmal over paroxysmal):Time (years)	0.01	[-0.02, 0.03]

Note. AF: Atrial fibrillation; CI: Confidence interval; GFR: Glomerular filtration rate; SF: Semantic Fluency Test, animals; TIA: Transient ischemic attack.

Table S31. Number (and percentage) of new cases of cognitive improvement greater than 1 SD per visit

	MoCA	SF	TMT-A	TMT-B	TMT B/A	DSST	CoCo
Follow-up 1	390 (14.60)	340 (12.72)	341 (12.76)	252 (9.43)	278 (10.40)	129 (4.83)	66 (2.47)
Follow-up 2	186 (6.96)	193 (7.22)	233 (8.72)	200 (7.49)	195 (7.30)	97 (3.63)	176 (6.59)
Follow-up 3	117 (4.38)	127 (4.75)	136 (5.09)	135 (5.05)	124 (4.64)	71 (2.66)	69 (2.58)
Follow-up 4	56 (2.10)	86 (3.22)	58 (2.17)	69 (2.58)	58 (2.17)	52 (1.95)	50 (1.87)

Note. CoCo: Cognitive construct; CI: Confidence interval; DSST: Digit Symbol Substitution Test; MoCA: Montreal Cognitive Assessment; SD: Standard deviation; SF: Semantic Fluency Test, animals; TMT-A: Trail Making Test A; TMT-B: Trail Making Test B; TMT B/A: ratio of Trail Making Test B/ Trail Making Test A.

Table S32. Estimates with 95% CI for MoCA with the depression as predictor of interest. The results for each covariate represent the effect after adjusting for all other variables in the model

	estimate	95 % CI
Depression (yes over no)	-0.22	[-0.36, -0.08]
Time (years)	0.09	[0.06, 0.12]
First visit (yes over no)	-0.09	[-0.13, -0.05]
Sex (female over male)	0.2	[0.12, 0.28]
History of diabetes (yes over no)	-0.31	[-0.41, -0.22]
Arterial hypertension (yes over no)	-0.08	[-0.16, -0.00]
History of stroke or TIA (yes over no)	-0.18	[-0.27, -0.10]
Oral anti-coagulant medication (yes over no)	0.07	[-0.05, 0.19]
Active smoker (yes over no)	-0.1	[-0.23, 0.04]
AF-type (non-paroxysmal over paroxysmal)	-0.08	[-0.15, -0.01]
GFR	0.02	[-0.00, 0.04]
Age at baseline (10 years)	-0.01	[-0.06, 0.04]
Depression (yes over no):Time (years)	-0.02	[-0.06, 0.03]

Note. AF: Atrial fibrillation; CI: Confidence interval; GFR: Glomerular filtration rate; MoCA: Montreal Cognitive Assessment; TIA: Transient ischemic attack.

Table S33. Estimates with 95% CI for MoCA with the history of diabetes as predictor of interest. The results for each covariate represent the effect after adjusting for all other variables in the model

	estimate	95 % CI
History of diabetes (yes over no)	-0.32	[-0.41, -0.22]
Time (years)	0.09	[0.06, 0.11]
First visit (yes over no)	-0.09	[-0.13, -0.05]
Sex (female over male)	0.2	[0.12, 0.28]
Depression (yes over no)	-0.24	[-0.37, -0.11]
Arterial hypertension (yes over no)	-0.08	[-0.16, -0.00]
History of stroke or TIA (yes over no)	-0.18	[-0.27, -0.10]
Oral anti-coagulant medication (yes over no)	0.07	[-0.05, 0.19]
Active smoker (yes over no)	-0.1	[-0.23, 0.04]
AF-type (non-paroxysmal over paroxysmal)	-0.08	[-0.15, -0.01]
GFR	0.02	[-0.00, 0.04]
Age at baseline (10 years)	-0.01	[-0.06, 0.04]
History of diabetes (yes over no):Time (years)	0	[-0.03, 0.03]

Note. AF: Atrial fibrillation; CI: Confidence interval; GFR: Glomerular filtration rate; MoCA: Montreal Cognitive Assessment; TIA: Transient ischemic attack.

Table S34. Estimates with 95% CI for MoCA with the history of stroke or TIA as predictor of interest. The results for each covariate represent the effect after adjusting for all other variables in the model

	estimate	95 % CI
History of stroke or TIA (yes over no)	-0.14	[-0.24, -0.05]
Time (years)	0.09	[0.06, 0.12]
First visit (yes over no)	-0.09	[-0.13, -0.05]
Sex (female over male)	0.2	[0.12, 0.28]
History of diabetes (yes over no)	-0.31	[-0.41, -0.22]
Arterial hypertension (yes over no)	-0.08	[-0.16, -0.00]
Depression (yes over no)	-0.24	[-0.37, -0.11]
Oral anti-coagulant medication (yes over no)	0.07	[-0.05, 0.19]
Active smoker (yes over no)	-0.1	[-0.23, 0.04]
AF-type (non-paroxysmal over paroxysmal)	-0.08	[-0.15, -0.01]
GFR	0.02	[-0.00, 0.04]
Age at baseline (10 years)	-0.01	[-0.06, 0.04]
History of stroke or TIA (yes over no):Time (years)	-0.04	[-0.07, -0.01]

Note. AF: Atrial fibrillation; CI: Confidence interval; GFR: Glomerular filtration rate; MoCA: Montreal Cognitive Assessment; TIA: Transient ischemic attack

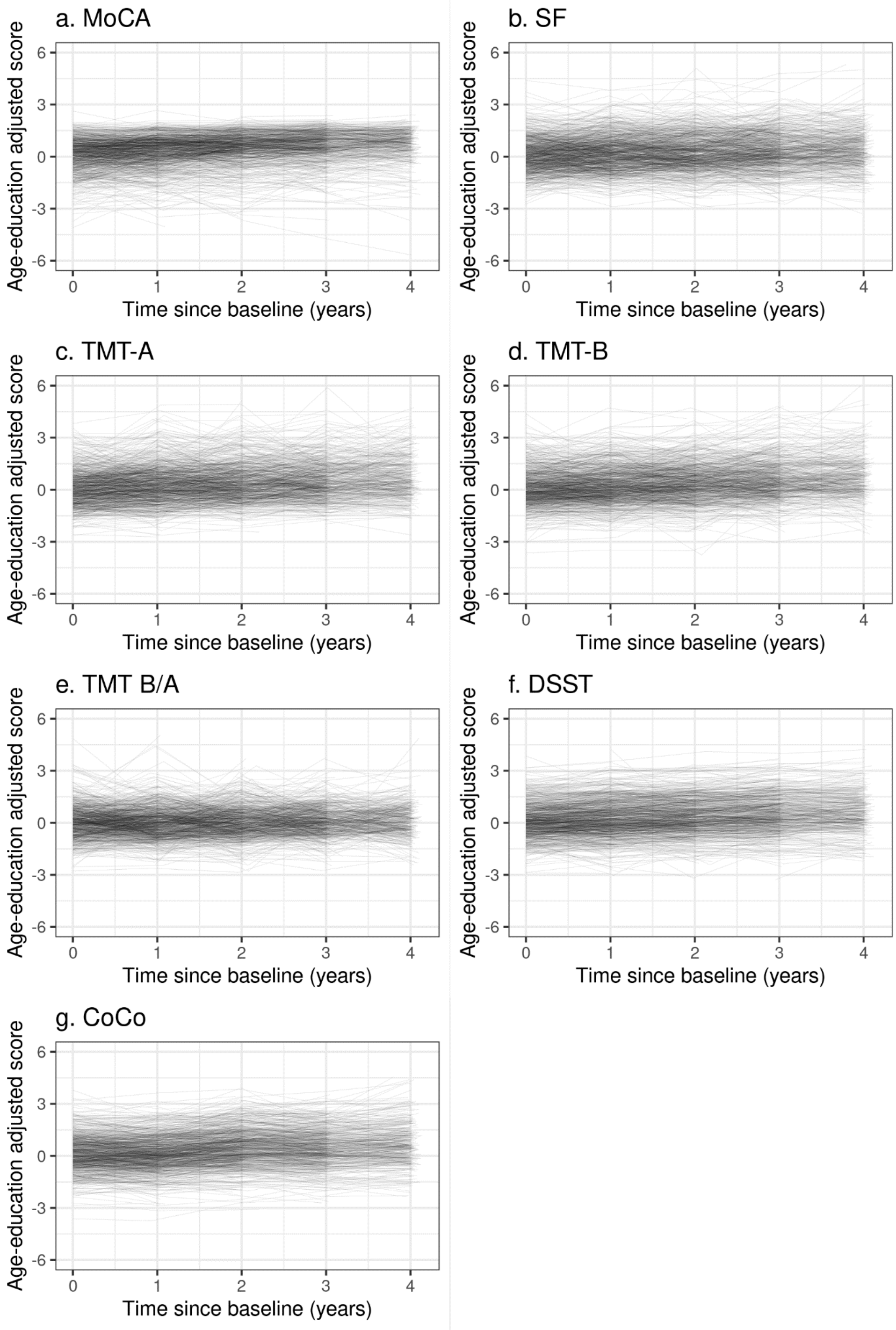

Figure S1 Spaghetti plots showing the evolution of the cognitive functioning until FU4 in patients with paroxysmal AF. CoCo: Cognitive construct; DSST: Digit Symbol Substitution Test; MoCA: Montreal Cognitive Assessment; SF: Semantic Fluency Test, animals; TMT-A: Trail Making Test A; TMT-B: Trail Making Test B; TMT B/A: ratio of Trail Making Test B/ Trail Making Test A.

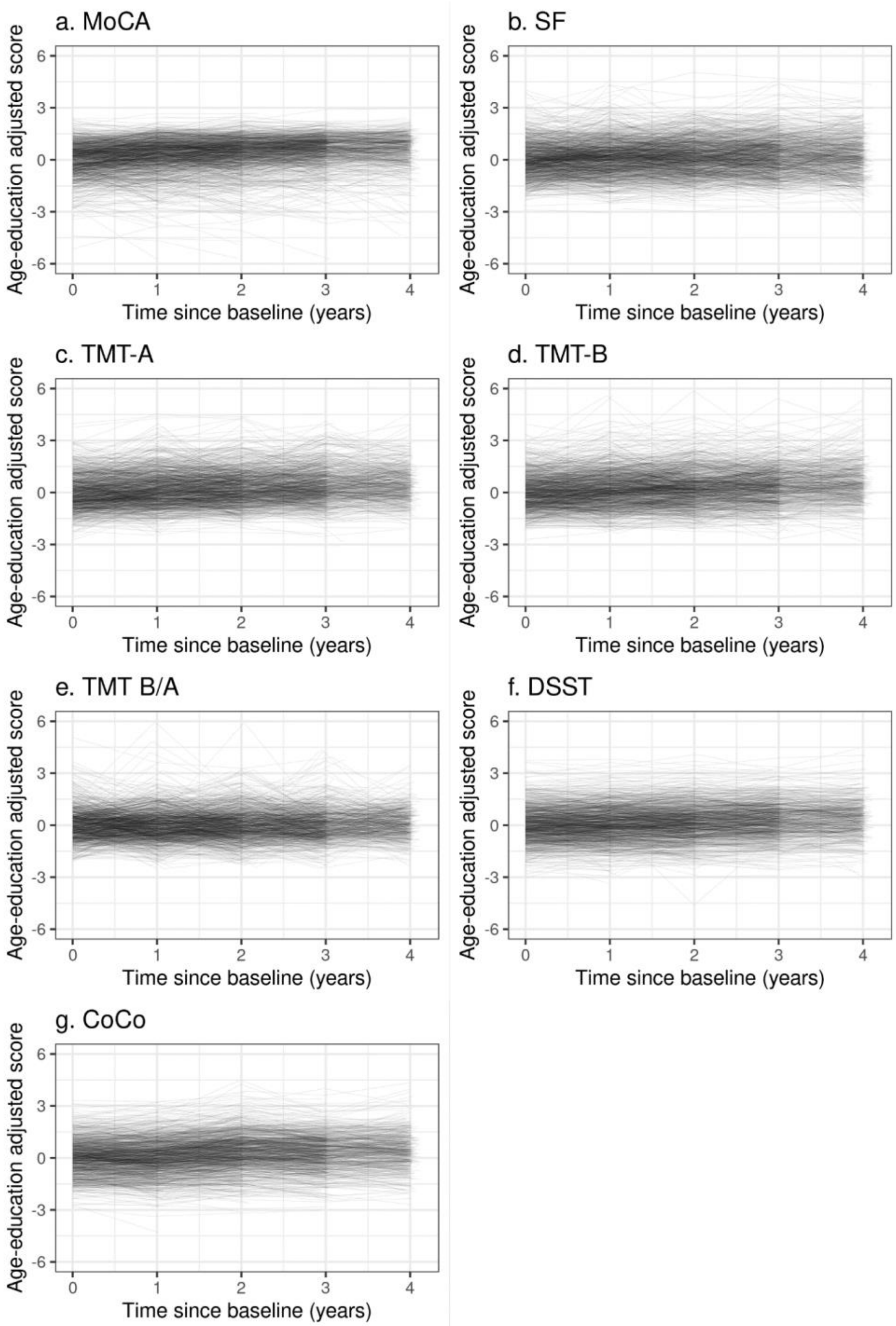

Figure S2 Spaghetti plots showing the evolution of the cognitive functioning until FU4 in patients with non-paroxysmal AF. CoCo: Cognitive construct; DSST: Digit Symbol Substitution Test; MoCA: Montreal Cognitive Assessment; SF: Semantic Fluency Test, animals; TMT-A: Trail Making Test A; TMT-B: Trail Making Test B; TMT B/A: ratio of Trail Making Test B/ Trail Making Test A.

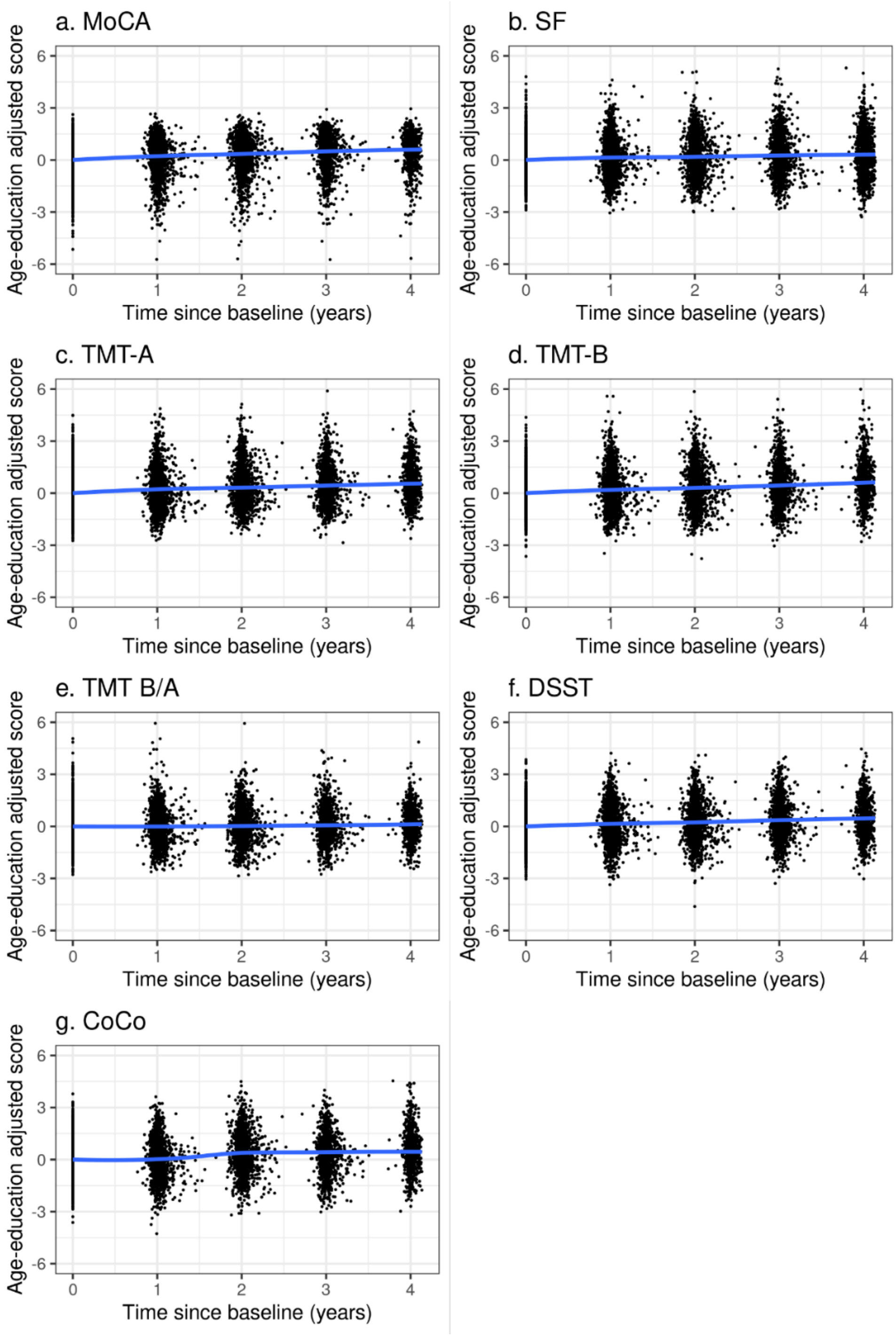

Figure S3 Scatter plot with spline to visualize the pattern of the cognitive functioning from baseline until FU4. CoCo: Cognitive construct; DSST: Digit Symbol Substitution Test; MoCA: Montreal Cognitive Assessment; SF: Semantic Fluency Test, animals; TMT-A: Trail Making Test A; TMT-B: Trail Making Test B; TMT B/A: ratio of Trail Making Test B/ Trail Making Test A.

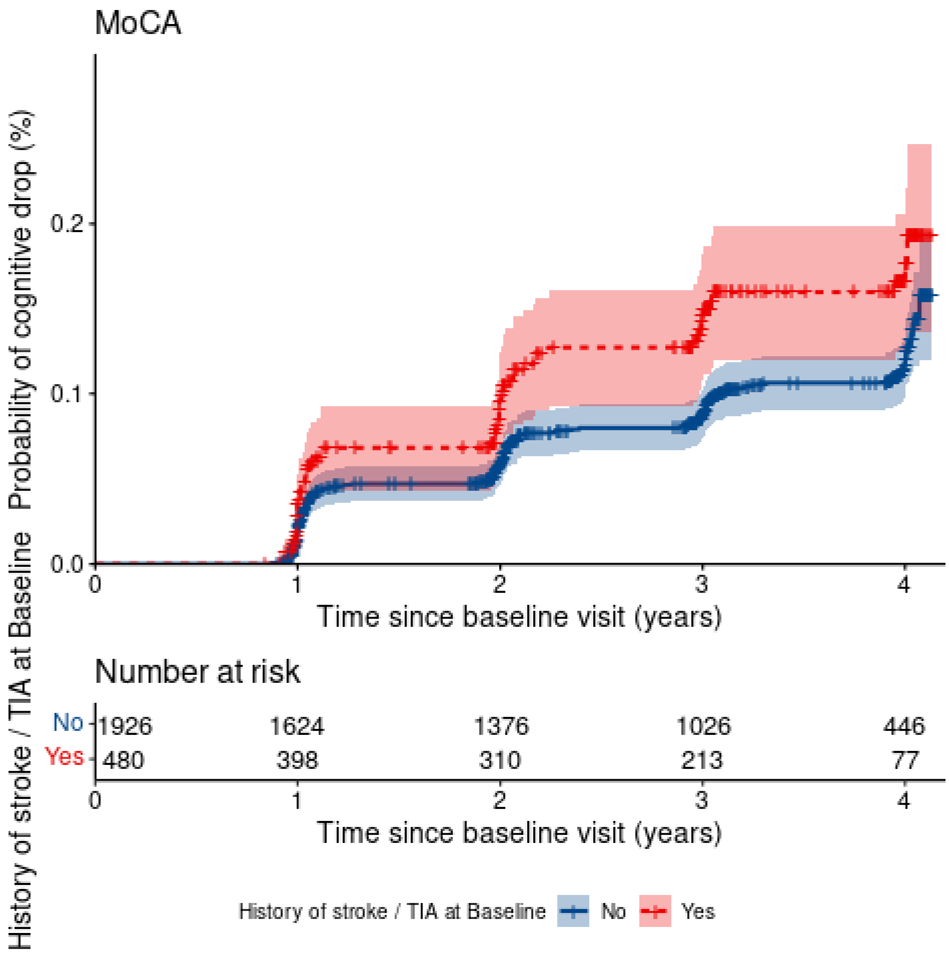

Figure S4 Cumulative probability of cognitive drop according to MoCA by history of stroke/ TIA. MoCA: Montreal Cognitive Assessment; TIA: Transient ischemic attack.

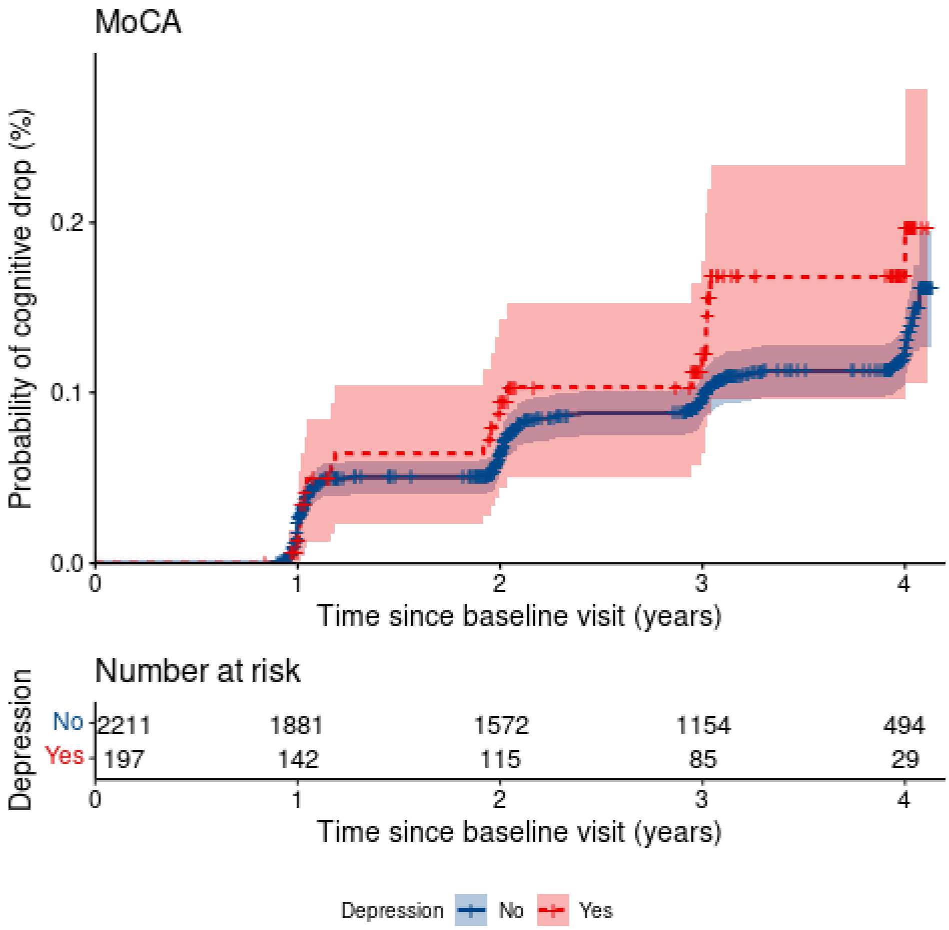

Figure S5 Cumulative probability of cognitive drop according to MoCA by depression. MoCA: Montreal Cognitive Assessment.

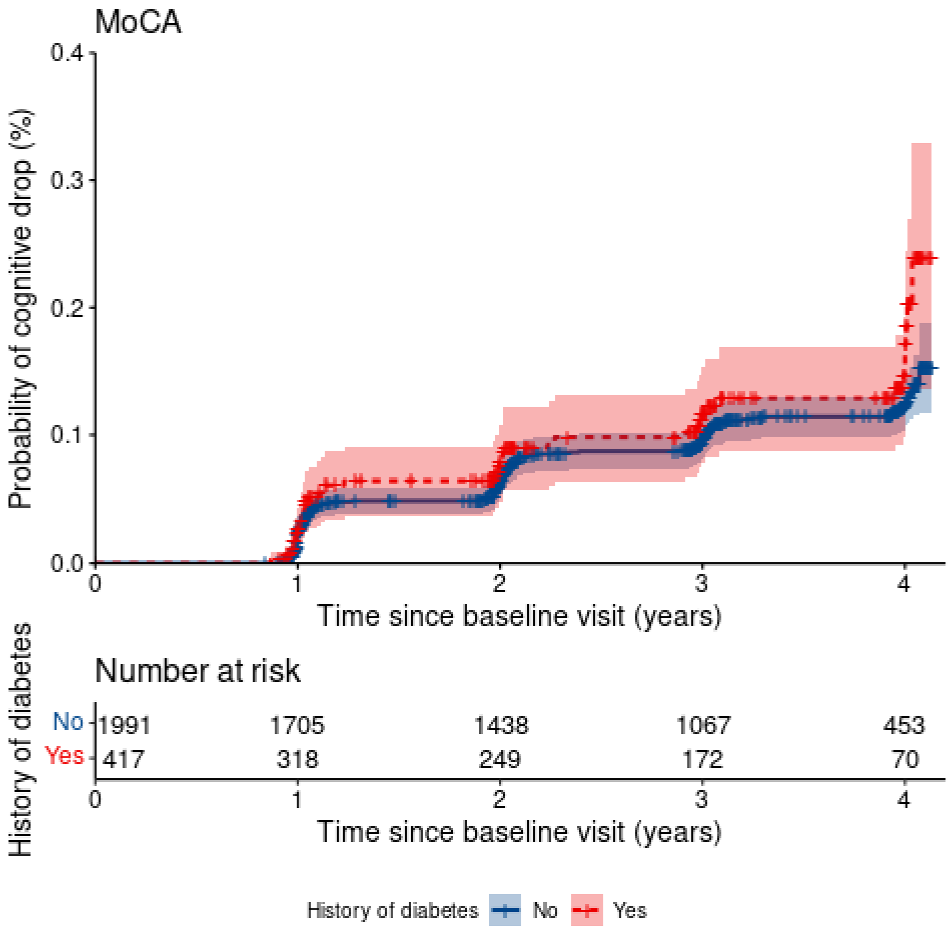

Figure S6 Cumulative probability of cognitive drop according to MoCA by history of diabetes. MoCA: Montreal Cognitive Assessment.
